# Low-density lipoprotein receptor-deficient hepatocytes differentiated from induced pluripotent stem cells allow familial hypercholesterolemia modeling, CRISPR/Cas-mediated genetic correction, and productive hepatitis C virus infection

**DOI:** 10.1186/s13287-019-1342-6

**Published:** 2019-07-29

**Authors:** Jérôme Caron, Véronique Pène, Laia Tolosa, Maxime Villaret, Eléanor Luce, Angélique Fourrier, Jean-Marie Heslan, Samir Saheb, Eric Bruckert, María José Gómez-Lechón, Tuan Huy Nguyen, Arielle R. Rosenberg, Anne Weber, Anne Dubart-Kupperschmitt

**Affiliations:** 1 INSERM UMR_S1193, Hôpital Paul Brousse, Villejuif, France; UMR-S1193, Université Paris-Saclay, Hôpital Paul Brousse, Villejuif, France; DHU Hepatinov, Hôpital Paul Brousse, Villejuif, France; 2 Université Paris Descartes, EA4474 Paris, France; 3 Unidad de Hepatología Experimental, Instituto de Investigación Sanitaria La Fe, Valencia, Spain; 4 Centre de Recherche en Transplantation et Immunologie UMR1064, INSERM, Université de Nantes, Nantes, France; 5 Service d’Endocrinologie Métabolisme, Hôpital Pitié-Salpêtrière, Paris, France; 6 AP-HP, Hôpital Cochin, Service de Virologie, Paris, France; 7 CIBERehd, FIS, Barcelona, Spain

**Keywords:** Cardiovascular disease, Genome editing, Cell models, Cell therapy, Gene therapy, Personalized medicine

## Abstract

**Background:**

Familial hypercholesterolemia type IIA (FH) is due to mutations in the low-density lipoprotein receptor (LDLR) resulting in elevated levels of low-density lipoprotein cholesterol (LDL-c) in plasma and in premature cardiovascular diseases. As hepatocytes are the only cells capable of metabolizing cholesterol, they are therefore the target cells for cell/gene therapy approaches in the treatment of lipid metabolism disorders. Furthermore, the LDLR has been reported to be involved in hepatitis C virus (HCV) entry into hepatocytes; however, its role in the virus infection cycle is still disputed.

**Methods:**

We generated induced pluripotent stem cells (iPSCs) from a homozygous LDLR-null FH-patient (FH-iPSCs). We constructed a correction cassette bearing LDLR cDNA under the control of human hepatic apolipoprotein A2 promoter that targets the adeno-associated virus integration site *AAVS1*. We differentiated both FH-iPSCs and corrected FH-iPSCs (corr-FH-iPSCs) into hepatocytes to study statin-mediated regulation of genes involved in cholesterol metabolism. Upon HCV particle inoculation, viral replication and production were quantified in these cells.

**Results:**

We showed that FH-iPSCs displayed the disease phenotype. Using homologous recombination mediated by the CRISPR/Cas9 system, FH-iPSCs were genetically corrected by the targeted integration of a correction cassette at the *AAVS1* locus. Both FH-iPSCs and corr-FH-iPSCs were then differentiated into functional polarized hepatocytes using a stepwise differentiation approach (FH-iHeps and corr-FH-iHeps). The correct insertion and expression of the correction cassette resulted in restoration of LDLR expression and function (LDL-c uptake) in corr-FH-iHeps. We next demonstrated that pravastatin treatment increased the expression of genes involved in cholesterol metabolism in both cell models. Moreover, LDLR expression and function were also enhanced in corr-FH-iHeps after pravastatin treatment. Finally, we demonstrated that both FH-iHeps and corr-FH-iHeps were as permissive to viral infection as primary human hepatocytes but that virus production in FH-iHeps was significantly decreased compared to corr-FH-iHeps, suggesting a role of the LDLR in HCV morphogenesis.

**Conclusions:**

Our work provides the first LDLR-null FH cell model and its corrected counterpart to study the regulation of cholesterol metabolism and host determinants of HCV life cycle, and a platform to screen drugs for treating dyslipidemia and HCV infection.

**Electronic supplementary material:**

The online version of this article (10.1186/s13287-019-1342-6) contains supplementary material, which is available to authorized users.

## Background

Familial hypercholesterolemia type IIA (FH) is an autosomal dominant inherited disorder of lipid/cholesterol metabolism due to mutations in the gene encoding the low-density lipoprotein receptor (LDLR). This disease is characterized by elevated plasma low-density lipoprotein-cholesterol (LDL-c) levels, which lead to severe premature cardiovascular diseases and early death [1]. FH is one of the most common genetic diseases, with a prevalence of 1/500 in its heterozygous form. This number might however be underestimated as recently, direct screening in a general Northern European population diagnosed approximately 1/200 patients with heterozygous FH [2]. Homozygous FH patients (1/10^6^) have very high plasmatic cholesterol levels (> 1000 mg/dL) and LDL-apheresis is the only available treatment to avoid heart failure and death occurring at an early age in absence of treatment [3, 4].

Hepatocytes play a central role in cholesterol and lipid homeostasis as they are the only cells in the organism able to regulate cholesterolemia by secreting cholesterol into the blood via lipoproteins and metabolizing it into bile acids. We and others previously showed that human induced pluripotent stem cells (hiPSC) derived from FH patients’ cells displayed impaired LDL-c internalization and that expression of a normal LDLR cDNA by mean of lentiviral or episomal vectors restored LDLR endocytosis. However, none of these iPSCs exhibited a homozygous null *LDLR* mutation [5–9].

The recent advancement of customized engineered endonucleases has demonstrated that CRISPR/Cas yielded efficient gene targeting in human iPSCs. A number of studies have used CRISPR/Cas9 in vitro and in vivo to explore various aspects of liver biology [10–13]. The most used safe harbor locus for gene targeting, where transgenes can be stably inserted and expressed from external promoters without showing any signs of genotoxic effects, is the Adeno-Associated Virus Integration Site 1 *(AAVS1)* locus [14].

The LDLR has been proposed to be one of the host factors participating in hepatitis C virus (HCV) entry into hepatocytes, although the role of this molecule in the virus infection cycle is still controversial [15–17]. The most widely used cells to culture HCV (Huh-7 cell line) [18] diverge significantly from differentiated hepatocytes. Primary human hepatocytes (PHHs) support the complete infection cycle including the production of workable titers of progeny virus, but their sources are limited [19]. Hepatocyte-like cells (HLCs) derived from iPSCs are permissive to HCV but the previously reported titers of progeny virus were very low, most likely attributable to their immature phenotype compared with PHHs [20].

Here, we report the first models of patient-specific FH-iPSCs with a homozygous null *LDLR* mutation and corrected counterparts via CRISPR/Cas9-mediated targeted insertion of an LDLR expression cassette at the safe harbor adeno-associated virus integration site *(AAVS1)*. We show that our differentiation process results in metabolically functional and polarized hepatocytes (iHeps). We demonstrate that our correcting LDLR cassette controlled by hepatic specific regulatory sequences of apolipoprotein A2 (*APOA2*) gene is specifically expressed in corrected hepatocytes (corr-FH-iHeps), restores LDLR function and is upregulated by pravastatin, increasing LDL-c uptake. Finally, we establish that iHeps support robust HCV production of infectious virus particles. In addition, this production was significantly lower in FH-iHeps compared to corr-FH-iHeps, indicating a role of the LDLR in HCV morphogenesis rather than in virus entry.

## Methods

### Generation of human iPSCs (hiPSCs) from a hypercholesterolemic patient’s fibroblasts

Eight-millimeter skin punch biopsy was obtained from a volunteering hypercholesterolemic (FH) patient homozygous for the g.10891 C>T (c.97C->T, p.Q12X) mutation leading to the absence of LDLR expression. Fibroblasts were derived from the donated tissue using standardized in house protocol and expanded in Dulbecco’s modified Eagle’s medium (DMEM) containing 1% non-essential amino acids (NEAA), 1% l-glutamine, 1% penicillin/streptomycin (all from Gibco) and 10% fetal bovine serum (PAA). Cells were passaged in 100 mm culture dishes (Corning, Corning Life Sciences, Dutscher) and were split 1:3 once they reached confluency.

FH-iPSCs were generated, amplified, and cryopreserved at passage 5 by Phenocell SAS (Evry, France) using non-integrative episomes [21] and their standard feeder-free routine.

### Culture of human iPSCs

hiPSCs were thawed onto irradiated mouse embryonic fibroblasts (MEF) feeder cells (Globalstem) at 3.5 × 10^5^ cells per 6 mm culture dish and cultured in hiPSC medium (DMEM, 20% knockout serum replacement (KOSR) (Gibco), 1% penicillin/streptomycin, 1% NEAA, 1% l-glutamine, 100 μM β-mercaptoethanol (Sigma), 10 ng/mL FGF2 (PeproTech)) in a 5% CO2 / 5% O2 incubator and medium was changed daily. Cells were then mechanically picked once a week and passaged onto new MEF-coated culture dishes. After 2 or 3 passages, colonies were mechanically picked and transferred onto Geltrex-coated culture dishes (Gibco) and cultured in StemMACS™ iPS-Brew XF medium (Miltenyi Biotec) changed every 2 days. Cells were mechanically passaged onto new Geltrex-coated dishes for further expansion of hiPSCs. To start a differentiation protocol, cells were dissociated into single-cell suspension by Cell Dissociation Buffer (CDB) (0.1 mg/ml EDTA, 0.5 mg/ml BSA in phosphate-buffered saline (PBS) 1×) for 5 min at 37 °C and were seeded in StemMACS™ iPS-Brew XF (40 000 cells per cm^2^) supplemented with 10 μM Rock inhibitor onto gelatin-coated plates.

### Targeted integration

The insertion, mediated by CRISPR/Cas9, of the “PuroR-normal human LDLR cDNA” expression cassette into *AAVS1* locus of hiPSCs, was performed by nucleofection (Amaxa Nucleofector, Lonza) of 3 plasmids respectively harboring the sgRNA *AAVS1* targeting sequence (GGGGCCACTAGGGACAGGAT), the spCas9 endonuclease [22] and a donor cassette containing *hAPOA2* promoter driving hLDLR cDNA expression cloned into pZDonor-*AAVS1* puromycin vector (Sigma-Aldrich). The expression cassette also contains Woodchuck hepatitis virus Posttranslational Regulatory Element (WPRE) sequence to improve protein expression by facilitating mRNA nuclear export. Cells were then seeded and incubated with increasing doses of puromycin (from 0.5 to 2 μg/mL) and resistant clones were isolated and further expanded for analysis and cryopreservation. Correct insertion of the expression cassette by homologous recombination was determined by PCR and Sanger sequencing with primers spanning the 5′ and 3′ homology arms, respectively (Table 1).

**Table 1:** Primers for *LDLR* Sanger sequencing and targeted insertion used in this study

Gene	Forward (5′–3′)	Reverse (5′–3′)	Annealing temp. (°C)
Primers used to screen FH mutation			
*LDLR* (exon 2)	TTTCCAGCTACGACACAGCAGGT	AGAACTGAGCAATCAAGCGGTTGA	55.8
Primers used for assessment of the targeted integration of the *LDLR* cassette			
5′ junction	CTGCCGTCTCTCTCCTGAGT (5′ arm)	GTGGGCTTGTACTCGGTCAT (Puro(R))	62
3′ junction	GTCAGCTCCTTTCCGGGACT (WPRE)	GGAACGGGGCTCAGTCTG (3′ arm)	62
*AAVS1* targeted site by CRISPR/CAS9	GCCCTATGTCCACTTCAGGA	ACAGGAGGTGGGGGTTAGAC	62

### Hepatocyte differentiation of iPSCs

In vitro differentiation of iPSCs into iHeps was performed using previously described protocols with modifications [23]. Briefly, 1 day before the passage of hiPSCs for differentiation, 12-well plates (Corning) were coated with 0.1% porcine gelatin (Sigma) for 1 h at room temperature, then with coating medium (DMEM, 10% fetal bovine serum (FBS, Hyclone), 1% non-essential amino acids, 1% penicillin/streptomycin and 1% l-glutamine), and incubated for 24 h at 37 °C. The following day, feeder-free hiPSCs were seeded as single cells on the coated plates in StemMACS™ iPS-Brew XF medium. Three days after, to initiate the differentiation into definitive endoderm, maintenance medium was replaced by RPMI (Gibco) and B27 serum-free supplement (Life Technologies), 1% non-essential amino acids, 1% penicillin/streptomycin and 1% l-glutamine and supplemented with 5 μM of CHIR99021 (Miltenyi Biotec) for 1 day followed by treatment with the same medium containing 100 ng/ml of Activin A (CellGenix) and 10 μM of LY294002 (VWR) for 4 days. For hepatic specification, 20 ng/ml FGF2 (CellGenix), 10 ng/ml BMP4 (R&D systems) and 50 ng/ml Activin A were added during 3 days. Then, to obtain hepatoblasts, RPMI without methionine (Gibco) was supplemented with 30 ng/ml FGF4 (R&D systems), 20 ng/ml HGF (Peprotech), 10 ng/ml EGF (Peprotech) for 2 days. At day 10, cells were passaged with CDB and seeded onto 12-well plates coated with 10 μg/mL fibronectin (Sigma) and 30 μg/mL of calf skin collagen type I (Sigma). Cells were then cultured in HPM medium (William’s E/Ham F12 1:1, 1% penicillin/streptomycin and 1% l-glutamine, 10^− 5^ M linoleic acid-Albumin (Sigma), 5.10^− 8^ M 3,3′,5-Triiodo-l-thyronine (Sigma), 0.07 IU Insulin (Actrapid® - Vidal), 6.10^− 4^ M vitamin C (Sigma), 6.10^−4^ M human apo-transferrin (Sigma), 1 mM sodium pyruvate (Gibco), 0,5 nM selenic acid (Sigma)) supplemented with 20 ng/mL HGF, 10 ng/mL oncostatin M (OSM, Peprotech) and 100 nM of dexamethasone (Sigma) for five days. For final maturation, OSM is removed and 0.5 μM of Compound E (Santa Cruz Biotechnology) and 5 μM of SB431542 (Tocris Biosciences) are added. The medium is changed every 2 days.

### Dil-LDL uptake

Cells were incubated with 1,1′-dioctadecyl-3,3,3,3′-tetramethylindocarbocyanine-perchlorate (Dil)-LDL (Bioquote Limited) at 2.5 μg/mL for 2 h. Then, cells were washed 3 times with cold PBS 1X, counterstained with DAPI and mounted in Fluoromount medium. Photographs were taken using TCS SP5 confocal microscope (Leica Microsystems).

For flow cytometry analyses, cells were dissociated for 5 min with Accutase (Fisher), harvested, centrifuged and pellets were suspended in coating medium and analyzed using LSR Fortessa flow cytometer (BD). Dil-LDL signal was collected by a phycoerythrin (PE) detector after transmission through a BP585/15 filter.

### Assessment of iHeps functionality

The indocyanin green (ICG) uptake test was assayed by incubating differentiated cells in medium supplemented with 0.5 mg/mL ICG for 60 min at 37 °C. Cells were then washed 3 times with PBS and fresh medium is added. ICG release was evaluated 4 h later.

Oil Red O staining was assayed by incubating fixed cells with 60% isopropanol (Sigma) for 5 min and then with 60% Oil Red O (Sigma). Cells were then washed 5 times with distilled water and counterstained with hematoxylin (Sigma) for 2 min before mounting and observed under an EVOS FL Auto Cell Imaging System (Life Technologies) microscope and treated with ImageJ software (http://imagej.nih.gov/ij/).

PAS staining was assayed by incubating fixed cells with 1% Periodic Acid Solution (Sigma) for 5 min. Cells were then washed 4 times with distilled water, incubated with Schiff’s reagent (Sigma) for 15 min, washed 3 more times and counterstained with hematoxylin for 2 min before mounting and analysis.

Formation of bile canaliculi was assayed by incubating differentiated cells in medium containing 2 μM of DCFA for 30 min. Cells were then washed with cold PBS and DCFA efflux from hepatocytes into canaliculi structures was analyzed by microscopy.

Amounts of albumin secreted into the medium were determined by the human albumin ELISA Quantitation Kit (Bethyl; http://www.bethyl.com) following manufacturer’s instructions. Levels of APOB100 secreted into the medium were quantified by using the total human ApoB ELISA assay from ALerCHEK, as previously described [24].

### HCV infection

A high-titer stock of JFH1-HCV [18] was produced in Huh-7.5.1 cells as described previously [19]. This viral stock was used to inoculate iHeps at day 18 of the differentiation protocol in 12-well plates at an MOI of 2 ffu per cell. The culture medium was replaced with the inoculum diluted in the smallest volume of fresh medium sufficient for covering the cells. After a 6-h incubation at 37 °C, the inoculum was removed, and cells were washed 3 times with PBS, then cultured in fresh medium for the indicated times post-inoculation. Where indicated, 500 nM of the HCV polymerase inhibitor sofosbuvir, or dimethylsufoxide as carrier control, were added to the medium. For the purpose of comparison, PHHs purchased from Biopredic (Rennes, France) were inoculated as described previously [19] with the same virus stock at the same MOI.

### Karyotyping analyses

Chromosome analyses were carried out by standard karyotyping on cultured cells, with standard procedures (RHG and GTG banding). For mitotic preparations, cells were cultured in StemMACS™ iPS-Brew XF supplemented with 0.02 mg/ml colchicine (Eurobio) for up to 2 h. The cells were harvested and incubated with a warm hypotonic solution of 0.075 M KCl for 20 min, then cells fixed several times in cold Carnoy’s fixative (methanol/acetic acid, 3:1).

### DNA sequencing

Genomic DNA was extracted using GeneJET Genomic DNA purification kit (ThermoFisher) according manufacturer’s instructions and amplicons of interest (Table 1) were purified using GeneJET Gel extraction kit (Fermentas). For the FH mutation, DNA sequencing was performed by Cochin Institute sequencing platform (Eurofins, France). Chromatograms were treated and analyzed by SeqScanner2 software. For the verification of the good insertion of the therapeutic cassette, 50 ng of genomic DNA was PCR amplified with pairs of primers overlapping either the 5′ junction or the 3′ junction of the *AVVS1* locus. PCR program consisted in hot start at 95 °C for 5 min followed by 35 cycles of 95 °C-10 s/62 °C-20 s/72 °C-45 s and 3 min 72 °C. PCR products were analyzed on a capillary electrophoresis device LabchipGX (5Kchip Caliper Lifesciences) and sequenced from both ends using each primer used for the PCR. Sequences were aligned and compared to the SA-2A-puro-pA donor plasmid for the joining regions of the LDLR cassette (Fig. 2).

### RNA extraction, RT-PCR and quantitative RT-PCR (qRT-PCR)

Total cell RNA was extracted using RNeasy Mini kit (Qiagen) following the manufacturer’s recommendations. The amount of isolated RNA was determined using UV-visible Nanodrop Lite (ThermoFisher). For RT-PCR, cDNAs were obtained by Superscript First-Strand Synthesis System (Invitrogen) and simple PCR assays were performed using Platinum Taq DNA polymerase (Invitrogen) according to the manufacturers’ instructions. For qRT-PCR, cDNAs were reverse transcribed by QuantiTect kit (Qiagen) and processed by real-time PCR over 40 cycles using Maxima SYBR Green/ROX qPCR master mix (ThermoFisher) and an ABI-7000 real-time PCR system and ABI 7000 software (Applied Biosystems). Three independent biological triplicates were analyzed. All samples were amplified in triplicates and changes in gene expression were analyzed using the ΔΔCt method by normalizing against RPL13a or TBP, RPLO, and h-PBGD. Primer details are shown in Table 2.

**Table 2:** RT-PCR and RT-qPCR primers used in this study

Gene	Forward (5′–3′)	Reverse (5′–3′)	Annealing temp. (°C)
Primers used for RT-PCR			
*OCT4*	GTGGAGGAAGCTGACAACAA	CAGGTTTTCTTTCCCTACGCT	54
*NANOG*	AGCTACAGCATGATGCAGGA	GGTCATGGAGTTGTACTGCA	54
*SOX2*	TCCAACATCCTGAACCTCAG	GACTGGATGTTCTGGGTCTG	55
*ALBUMIN*	CCTTTGGCACAATGAAGTGGGTAACC	CAGCAGTCAGCCATTCACCATAGG	58
*A1AT*	CCAACAGCACCAATATCCATCTTC	GTCCTCTTCCTCGGTGTCCTTG	60
*HNF1α*	AGACTTCACGCCACCCATCCTC	TTCCTCCGCCCCTTCTTGGTTG	60
*ASGR*	TAGGAGCCAAGCTGGAGAAA	ACCTGCAGGCAGAAGTCATC	56
*ACTIN*	GCACTCTTCCAGCCTTCCTTCC	CTGCTGTCACCTTCACCGTTCC	60
*FOXA2*	GCGACCCCAAGACCTACAG	GGTTCTGCCGGTAGAAGGG	60
*GATA4*	CTAGACCGTGGGTTTTGCAT	TGGGTTAAGTGCCCCTGTAG	56
*HNF4α*	CGGGTGTCCATACGCATCCTTG	GACCCTCCCAGCAGCATCTCCT	60
Primers used for RT-qPCR			
*CYP2D6*	AAGTACAGGGCTTCCGCATCC	GGGCTCACCAGGAAAGCAA	60
*CYP3A4*	AGAAAGTCGCCTCGAAGATACAC	CAGAGCTTTGTGGGCTCAGTT	56
*CYP2A6*	GCCCGCAGAGTCAAAAAGGA	CCCCTTCTCATTCAGGAAGTGCT	57
*SREBP2*	CTCCATTGACTCTGAGCCAGGA	GAATCCGTGAGCGGTCTACCAT	60
*HMGCR*	CACGATGCATAGCCATCCTG	GTGCTTGCTCTGGAAAGGTC	64
*PCSK9*	TGTATGGTCAGCACACTCGG	TAGACACCCTCACCCCCAAA	62
*APOA1*	AGGCTCGGCATTTCTGGCAG	CGCTGTCCCAGTTGTCAAGG	59
*APOB*	AGCGTTCACCGATCTCCATCTG	TCAGATTCCCGGACC CTCAACT	59
*APOC3*	GTTACATGAAGCACGCCACC	CTCGCAGGATGGATAGGCAG	59
*APOE*	TGGGTCGCTTTTGGGATTAC	AGTTGTTCCTCCAGTTCCGA	59

### Immunocytochemistry

Cells were fixed with 4% Paraformaldehyde (Euromedex) for 15 min at room temperature, then permeabilized with 0.5% Triton X-100 for 20 min and blocked in 3% bovine serum albumin (BSA)-PBS for 30 min at room temperature. Primary antibodies were diluted in PBS, 1% BSA and incubated overnight at 4 °C. Secondary antibodies were diluted in PBS, 1% BSA and incubated for 1 h at room temperature. Cell nuclei were counterstained with DAPI and cells were mounted in Fluoromount medium (Sigma) (See primary and secondary antibodies’ dilutions in Table 3). For membrane proteins staining, fixed cells were blocked in PFS buffer (0,025% saponin (Fisher), 0,02% sodium azide (Sigma), 0.7% fish gelatin (Sigma) in PBS) for 30 min at 37 °C. Primary antibodies were diluted in PFS and incubated overnight at 4 °C. Secondary antibodies and DAPI were diluted into PFS and were incubated at 37 °C for 1 h and 5 min, respectively. Photographs were captured with a Leica HMR microscope (Leica Microsystems) or EVOS FL Auto Cell Imaging System (Life Technologies) and treated with ImageJ software (http://iagej.nih.gov/ij/).

**Table 3:** Primary and secondary antibodies used in this study

Cat. No	Company	Antibody	Species	Dilution
Antibodies used for immunofluorescence				
SC-5279	Santa Cruz Biotechnology	OCT4	Mouse	1/200
AF1997	R&D	NANOG	Goat	1/100
MAB4303	Millipore	SSEA-3	Mouse	1/200
MAB4360	Millipore	TRA-1-60	Mouse	1/200
SC-1237	Santa Cruz Biotechnology	GATA4	Goat	1/200
SC-6554	Santa Cruz Biotechnology	FOXA2	Goat	1/200
SC-8987	Santa Cruz Biotechnology	HNF4α	Rabbit	1/200
M0888	Dako	CK19	Mouse	1/200
SC-8399	Santa Cruz Biotechnology	AFP	Mouse	1/200
A6084	Sigma	ALB	Mouse	1/100
SC-27639	Santa Cruz Biotechnology	CYP3A4	Goat	1/100
A0012	Dako	A1AT	Rabbit	1/400
187362	Life Technologies	CLDN1	Rabbit	1/200
555675	BD Pharmingen	CD81	Mouse	1/200
NB400-131	Novus Biologicals	SRB1	Goat	1/200
NBP1-85047	Novus Biologicals	ZO-1	Rabbit	1/400
AB42488	Abcam	ASGR	Rabbit	1/100
SC-7258	Santa Cruz Biotechnology	Connexin 32	Goat	1/100
M3612	Dako	E-cadherin	Mouse	1/100
AB28482	Abcam	SREBP2	Rabbit	1/100
A12379	Molecular Probes	F-Actin	Mouse	1/200
AB5790	Abcam	PAX6	Rabbit	1/200
AB78078	Abcam	TUJ1	Mouse	1/100
AB5694	Abcam	αSMA	Rabbit	1/100
A11055 - A11057	Molecular Probes	Anti-goat	Donkey	1/1000
A32306 - A10042	Molecular Probes	Anti-rabbit	Donkey	1/1000
A21202 - A10037	Molecular Probes	Anti-mouse	Donkey	1/1000
Antibodies used for western blot				
AB52818	Abcam	LDLR	Rabbit	1/500
AB8227	Abcam	Actin	Rabbit	1/3000
NA934V	Thermo Fisher	Anti-Rabbit HRP	Donkey	1/10000
Antibodies used for flow cytometry				
12-8843-42	eBioscience	SSEA-4	Mouse	1/25
12-8883-82	eBioscience	TRA-1-81	Mouse	1/25
130-109-844	Miltenyi Biotec	CXCR4	Mouse	1/25
130-080-301	Miltenyi Biotec	EPCAM	Mouse	1/20
12-4742-42	eBioscience	Mouse IgG3-PE	Mouse	1/25
12-5890-82	eBioscience	Mouse IgM-PE	Mouse	1/25
130-091-836	Miltenyi Biotec	Mouse IgG2a-APC	Mouse	1/25
A07795	Beckman Coulter	Mouse IgG1-FITC	Mouse	1/20

### Flow cytometry

Cells were dissociated with CDB and suspended in PBS, 3% BSA. They were then incubated with conjugated antibodies or with control isotypes for 30 min at 4 °C in the dark. Cells were then washed with PBS, centrifuged and suspended in PBS, 1% BSA for analysis. Cells were detected with an Accuri C6 flow cytometer (BD biosciences). Dead cells were excluded from the analysis based on 7-Amino Actinomycin D (7-AAD) staining (Beckman Coulter). FACS antibodies and their dilutions are listed in Table 3.

### Western blot analyses

Total proteins were extracted using RIPA buffer (50 mM Tris-HCl, 150 mM NaCl, 1% Triton 100X, 1% sodium deoxycholate (all from Sigma) in water) with the addition of protease inhibitor cocktail (Roche). Thirty micrograms of total protein extracts was electrophoresed on an 8% acrylamide-SDS gel then transferred to a PVDF membrane (GE healthcare). The membrane was then blocked with PBS supplemented with 5% (w/v) dry milk and 0.1% Tween-20, washed with PBS containing 0.1% Tween-20 (PBST) and then incubated with primary antibodies diluted in PBS-milk-0.1% Tween-20 at 4 °C overnight. After washing in PBST, the membranes were incubated with horseradish peroxidase-conjugated secondary antibodies at room temperature for 1 h. Clarity Western ECL Substrate kit (Biorad) was used for the revelation and pictures were captured using a Fusion Solo Camera and Fusion software. Protein expression was quantified using ImageJ software with β-actin as a control for protein amount. Dilutions of primary and secondary antibodies are described in Table 3.

### Virological analyses

All analyses were performed at day 3 post-inoculation except for the assessment of HCV genome replication, which was done at day 1 post-inoculation, i.e., under conditions of a single infection cycle. Indeed, at later time points, multiple rounds of infection are expected to cause a bias in the assessment of viral genome replication per se if the production of viral particles is affected [24].

iHeps at day 18 of the differentiation protocol or PHHs were inoculated with JFH1-HCV at an MOI of 2. To assess HCV genome replication, Cells were lysed at day 1 post-inoculation and intracellular amounts of negative-strand HCV RNA were quantified by a strand-specific qRT-PCR technique described previously [25].

To assess production of total HCV particles, HCV RNA levels in filtered culture supernatants were collected at day 3 post-inoculation and were quantified by qRT-PCR using the m2000sp and m2000rt instruments and RealTime HCV kit (Abbott Diagnostics), a commercial standardized assay used for viral load quantification in clinical practice [26], as previously described [19].

To assess the production of infectious HCV particles, infectious titers in filtered culture supernatants collected at day 3 post-inoculation were determined by focus-formation assay, a classic titration assay based on inoculation of naive Huh-7.5.1 cells followed by detection of foci of infected cells by immunofluorescence, as described previously [19, 27].

### Statistics

All experiments were performed with at least 3 biologic replicates. Histograms and summary statistics reflect mean ± standard error of the mean. Unpaired Student’s t test was used for single comparisons. In all cases, statistical significance was denoted by (*) for *P* ≤ 0.05, by (**) for *P* ≤ 0.01 and by (***) for *P* ≤ 0.001.

## Results

### Patient-specific iPSCs display an FH phenotype in vitro

**Fig. 1:**
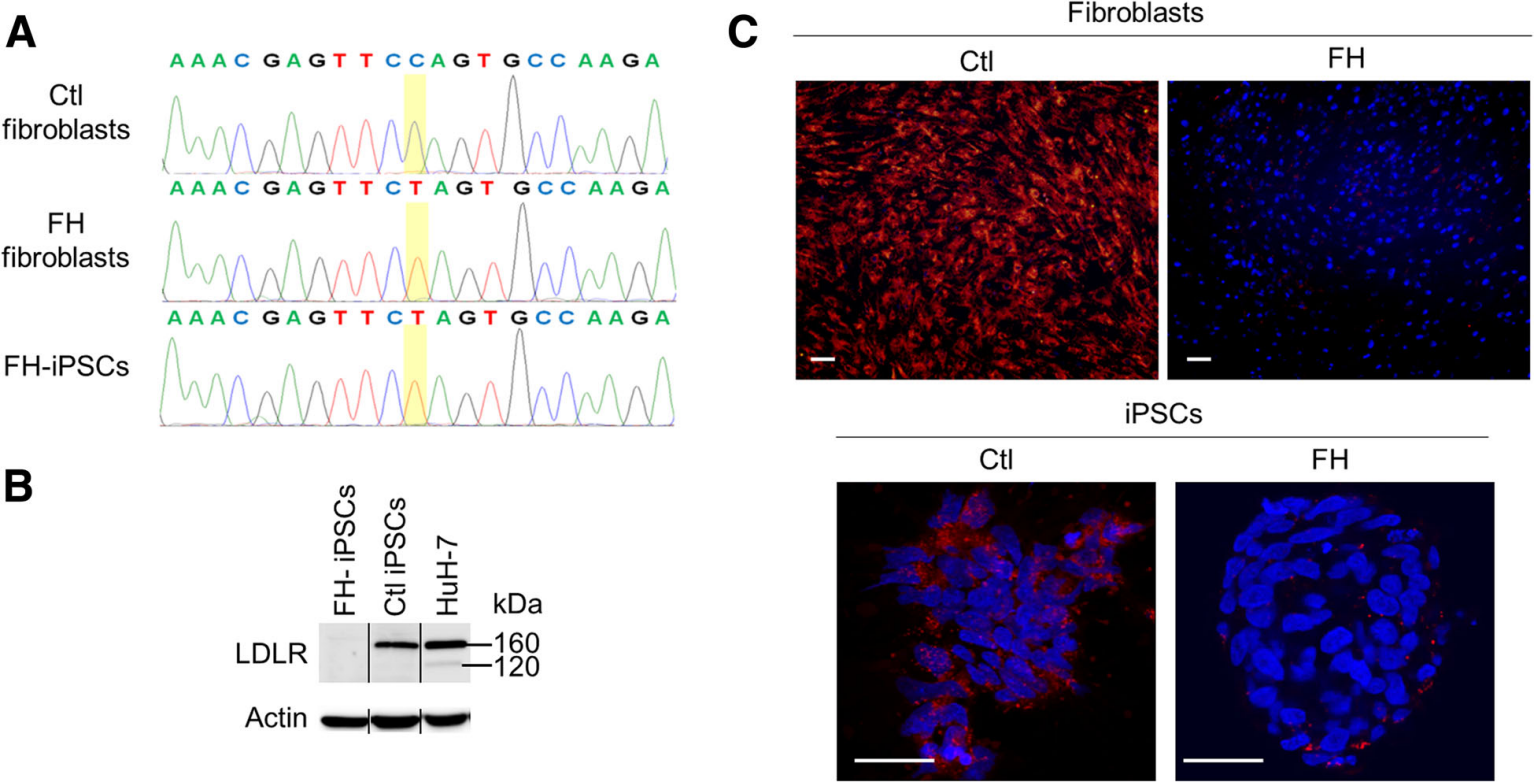
FH-iPSCs recapitulate FH phenotype in vitro. **a** DNA sequences depicting the mutated nucleotide g.10891C>T (c.97C->T, p.Q12X) in the *LDLR* exon 2 in FH-patient’s fibroblasts and iPSCs by contrast to normal human fibroblasts (Ctl). **b** Western blot analysis confirming the absence of LDLR protein in FH-iPSCs. **c** Representative confocal pictures of Dil-LDL internalization in ctl and FH-fibroblasts and in ctl and FH-iPSCs. Nuclei were counterstained with DAPI. Scale bar 50 μm

Clonal iPSC lines from a homozygous FH patient (FH-iPSC) carrying a nonsense mutation at codon 12 were generated by reprogramming skin fibroblasts and displayed hallmarks of pluripotency, stemness and normal karyotype (Additional file 1: Figure S1). Next, we sequenced exon 2 of the *LDLR* gene that revealed that the c.97C->T *LDLR* mutation present in the patient’s fibroblasts, resulting in a premature stop codon in the protein sequence (p.Q12X), was also found in FH-iPSCs (Fig. 1a). Accordingly, LDLR protein was not detected by western blot in FH-iPSCs by contrast with normal iPSCs (Fig. 1b) and LDL-cholesterol uptake was impaired in the patient’s fibroblasts and iPSCs compared to their respective controls, as assessed by internalization of a fluorescent LDLR ligand, 1,1′-dioctadecyl-3,3,3,3′-tetramethylindocarbocyanineperchlorate (Dil-LDL) (Fig. 1c).

### Genetic correction of LDLR defect by using CRISPR/Cas9 genome engineering

**Fig. 2:**
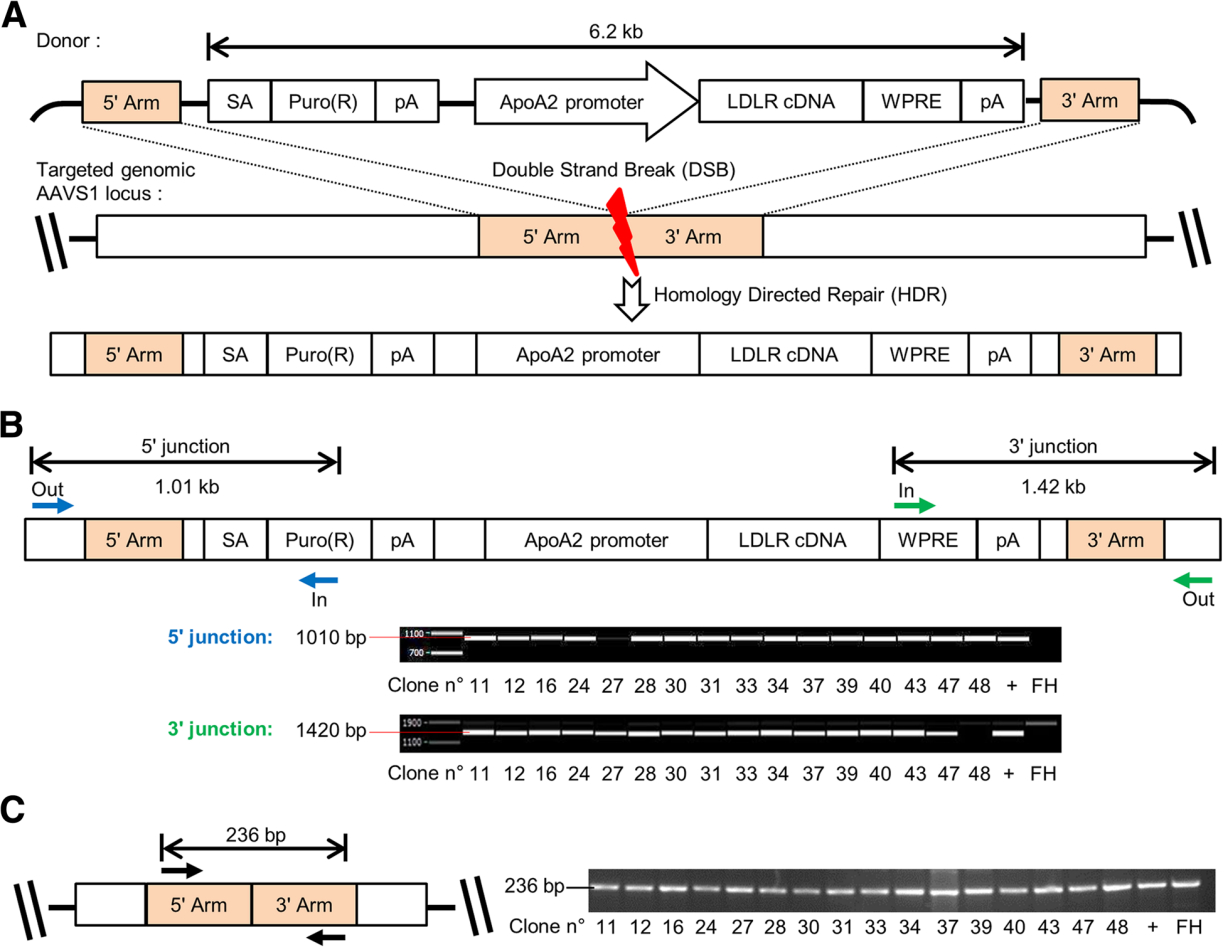
CRISPR/Cas9-mediated insertion of LDLR expression cassette at the *AAVS1* genomic site. **a** Design of the LDLR expression cassette in the AAVS1 SA-2A-puro-pA donor plasmid that contains sequences homologous to the sequences flanking the *AAVS1* double strand break (DSB) induced by CRISPR/Cas9 (5′ and 3′ Arms). Abbreviations: AAVS1, adeno-associated virus integration site 1, *APOA2*, apolipoprotein A2; pA, polyadenylation tail; Puro(R), puromycin resistance gene; SA, splice acceptor site; WPRE, woodchuck posttranslational regulatory element. **b** Junction PCR analyses showing correct integration of the cassette in 15/16 FH-iPSC clones. **c** PCR analysis corresponding to *AAVS1* targeted locus, showing absence of integration of the correcting cassette in one of the alleles

We then explored the potential of the CRISPR/Cas9 site-specific nuclease system to target the insertion of a correction cassette into the *AAVS1* safe harbor locus. We designed and constructed an expression cassette that harbors the entire wild-type LDLR cDNA under the control of 900 bp of the hepatic-specific apolipoprotein A2 *(APOA2)* gene regulatory sequences (Fig. 2a). It also contains a splice acceptor site allowing the puromycin resistance cDNA to be correctly spliced and translated when placed under the control of the endogenous promoter of the protein phosphatase 1 regulatory subunit 12C (*PPP1R12C*) gene, located at the *AAVS1* locus after targeted insertion. This therapeutic cassette, flanked in 5′ and 3′ by around 500 bp homology arms, as well as plasmids bearing the CRISPR/Cas9 system, were transfected into single-cell iPSCs. iPSCs were then seeded and incubated with increasing doses of puromycin and 34 resistant colonies were cloned. Junction PCR analyses on genomic DNA using primers spanning the 5′ and 3′ homology arms showed that 45% of the puromycin-resistant iPSC clones (15/34) were accurately integrated (Fig. 2b). The puromycin-resistance of the other clones may be due to an incorrect integration of the 3′ arm during the homologous recombination (as clone 48 in Fig. 2b), a bystander effect or an off-target integration of the therapeutic cassette. In addition, an endogenous *AAVS1* allele was detected in these 15 clones, indicating a monoallellic integration of the cassette (Fig. 2c). As homologous recombination (HR) usually occurs at a very low frequency, we were not able to screen a clone with an integration of the therapeutic cassette on both alleles of the *AAVS1* locus. One of these corrected FH-iPSCs (corr-FH-iPSCs) clones was chosen for further characterization that confirmed stemness, normal karyotype, and absence of any mutation in the inserted LDLR cDNA (data not shown). Furthermore, to confirm that this clone had been subjected to proper HR, we sequenced its 2 homology arms where the homologous recombination occurred and we did not detect any nucleotide insertion or deletion by comparison with human consensus sequence GRCh38 (data not shown). Of note, when we compared these sequences with the plasmid used for HR, we detected a T/A point mutation on the 5′ arm which is present in the plasmid but not in the consensus sequence, suggesting that the HR occurred downstream this position. (Additional file 2: Figure S2).

### Genetic correction rescues FH phenotype

We next used our previously described approach [23] to differentiate diseased, corrected and normal iPSCs into definitive endoderm and hepatoblasts. RNA analyses showed that, in the course of differentiation, cells sequentially expressed stage-specific markers (Additional file 3: Figure S3). Our differentiation process resulted in homogenous populations that expressed high levels of specific markers such as GATA4, FOXA2 or CXCR4 and EPCAM, respectively as shown by immunofluorescence studies. Moreover, as the hepatoblasts are committed bipotent hepatic progenitors, they co-expressed hepatocytic (hepatic nuclear transcription factor, HNF4α) and biliary (cytokeratin 19, CK19) markers (Additional file 4: Figure S4).

**Fig. 3:**
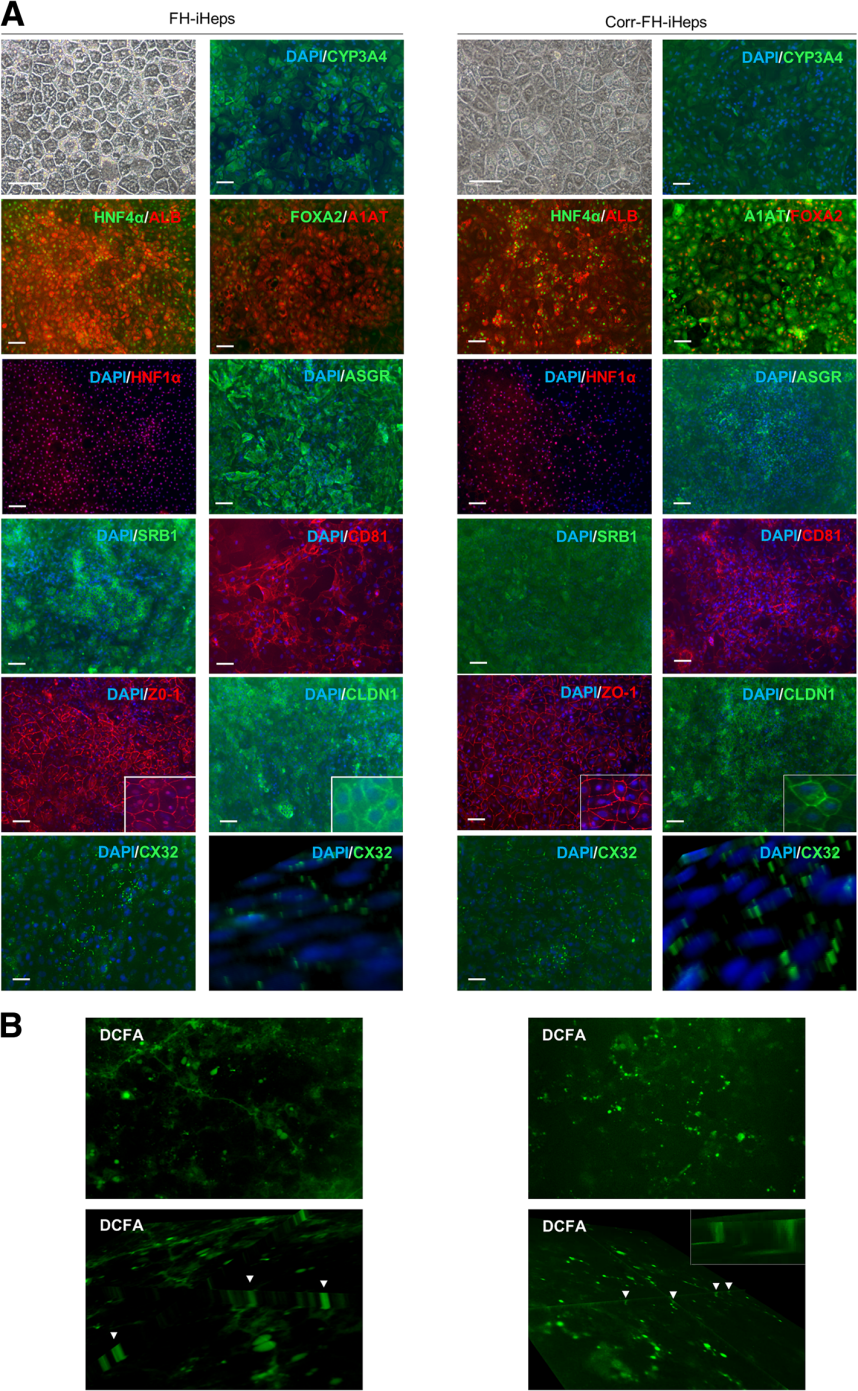
Differentiation of FH- and corr-FH-iPSCs into hepatocytes. **a** Representative pictures of cell morphology and immunostainings of the indicated markers at day 25 of differentiation. Scale bars: 50 μm. **b** Representative images of DCFA excretion at the biliary poles of corr-FH-iHeps. All images were taken with × 10 objective, z-stacks of xy sections of the cells were acquired with an epifluorescence microscope (Nikon Elipse) and analyzed with ImageJ software. Arrowheads indicate bile canaliculi

Despite significant improvements in protocols for generating hepatocyte-like cells from either hESCs or hiPSCs, generated hepatocytes still exhibit an immature phenotype [28–31]. Based on recent studies, we therefore refined our differentiation protocol to differentiate our hepatoblasts into more mature hepatocytes (iHeps) [31] (Additional file 3: Figure S3). The morphology of our resulting iHeps closely resembled that of PHHs, with the presence of two nuclei in some cells and most cells positively stained for asialoglycoprotein receptor (ASGR), CYP3A4 and albumin. They also expressed molecules used as co-receptors for HCV entry, including scavenger receptor B1 (SRB1) and CD81 (Fig. 3a, Additional file 5: Figure S5). Importantly, iHeps showed signs of epithelial polarity, a fundamental feature required for structural and functional integrity, as shown by membrane immunofluorescence staining for the apical marker tight junction protein ZO-1 and claudin-1 (Fig. 3a). They also expressed punctiform connexin 32 (CX32), the main gap junction protein expressed in normal PHHs [32] and gap junction plaques between adjacent cells were visible by z-stack reconstruction (Fig. 3a). 5(6)-Carboxy-2′,7′-dichlorofluorescein diacetate (DCFA) allows to examine biliary efflux through multidrug resistance protein transporter 2, and cell polarity. CDF, DCFA derivative excreted into the bile canaliculi, was visible in the usual top view and z-stack reconstruction, showing the functionality of the bile canaliculi in our differentiated iHeps (Fig. 3b). They also displayed the expression of different apolipoproteins and of three of the major cytochromes P450 (*CYP3A4*, *CYP2A6,* and *CYP2D6*) such as physiological hepatocyte functions including albumin secretion, lipid and glycogen storage as well as uptake of indocyanin green (ICG) and its subsequent excretion (Additional file 6: Figure S6). Collectively, these results suggest that the cells are moving towards a functional mature hepatocyte state, i.e., a differentiation step further compared to previously reported phenotypes [29].

**Fig. 4:**
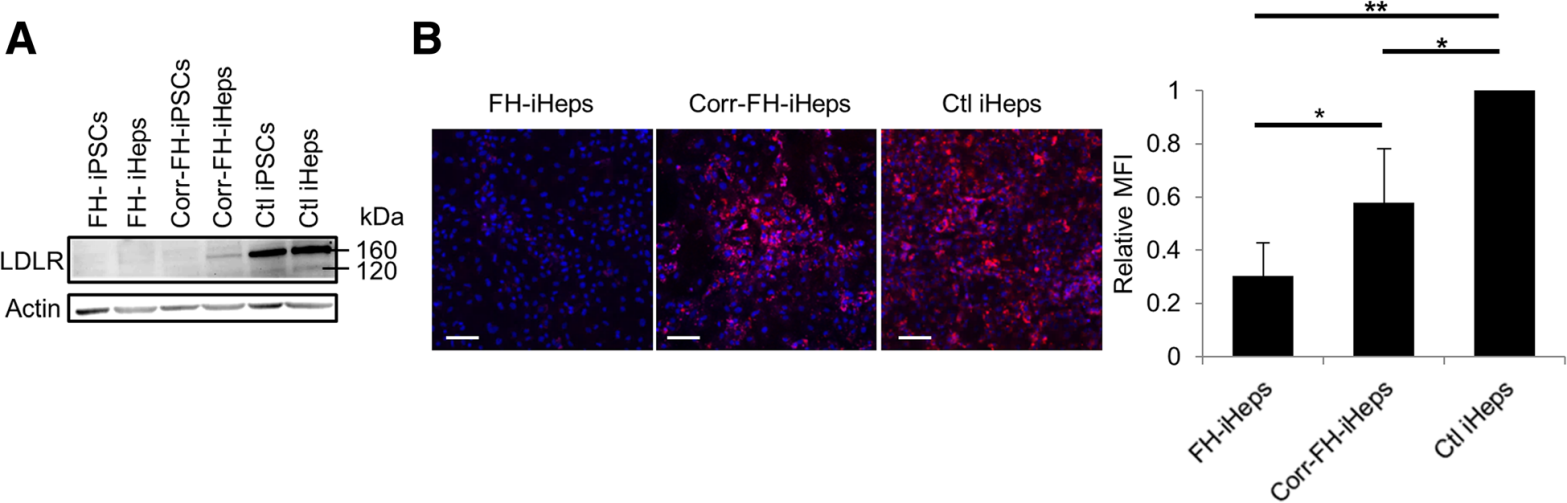
Phenotypic rescue of corr-FH-iHeps. **a** Western blot analysis demonstrating the specificity of *APOA2* promoter in corr-FH-iHeps. **b** Representative confocal images of Dil-LDL internalization in FH-iHeps, corr-FH-iHeps and Ctl iHeps. Nuclei were counterstained with DAPI. Scale bars: 100 μm. FACS analysis of internalized Dil-LDL levels: results are expressed as mean fluorescence intensity (MFI) relative to Ctl iHeps (*n* = 3). Statistical significance was determined by Student’s test, *P < 0.05, **P < 0.01

We next looked for the restoration of a functional phenotype in corr-FH-iHeps. LDLR protein was absent in both FH-iPSCs and FH-iHeps as assessed by western blot. On the contrary, it was detected in corr-FH-iHeps but not in corr-FH-iPSCs by contrast with control iPSCs, confirming that the hepatic specificity of the *APOA2* promoter was maintained in our model (Fig. 4a). Dil-LDL was detected by flow cytometry in corr-FH-iHeps at a level corresponding to 57% of control iHeps, consistent with a monoallelic insertion of the therapeutic cassette inside the *AAVS1* locus (Fig. 4b). As expected, a remaining detection of LDLc was observed in FH-iHeps, due to a passive endocytosis. This other LDL-c-mediated entry is independent of the LDLR and non-saturable, explaining this detection [33].

### Differentiated iHeps recapitulate the regulation of genes involved in cholesterol metabolism

We then studied the regulation by statins of three genes, sterol response element-binding protein 2 (*SREBP2*), 3-hydroxy-3-méthyl-glutaryl-coenzyme A reductase (*HMGCR*), and proprotein convertase subtilisin kexin/type 9 (*PCSK9*), which encode major regulators of cholesterol level in hepatocytes that affect *LDLR* expression level. Statins are routed primarily to the liver where they bind and inhibit HMGCR, the rate-limiting enzyme in cholesterol biosynthesis, resulting in its decrease in hepatocytes. Consequently, expression of the *LDLR* gene and LDL uptake are enhanced via SREBP2 activation [1] which results in translocation of its active form from the endoplasmic reticulum to the nucleus. There, it binds to regulatory sequences known as sterol regulatory elements (SRE), located in the promoters of SREBP2 target genes, mainly involved in cholesterol regulation, such as *LDLR*, *HMGCR* and *PCSK9*. The latter encodes a protein that is a natural inhibitor of the LDLR by directing it to lysosomes for degradation [34]. In corr-FH-iPSCs, statins are also expected to enhance the transcription of LDLR cDNA, controlled in our cassette by SRE-containing *APOA2* regulatory sequences [35].

**Fig. 5:**
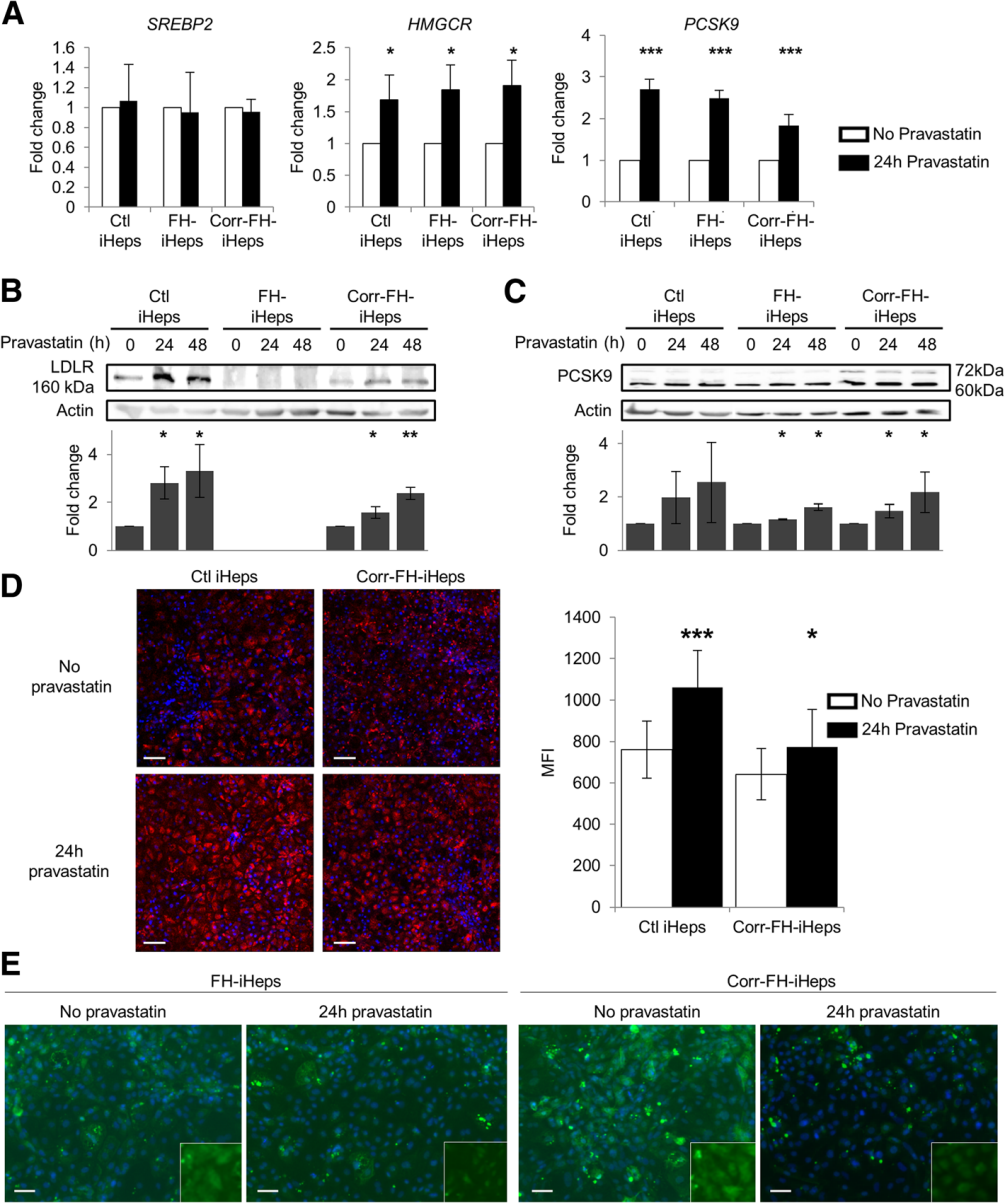
Effect of pravastatin on the regulation of target genes involved in cholesterol metabolism. **a** Quantitative reverse-transcription PCR (qRT-PCR) analyses of the expression of SREBP2, HMGCR and PCSK9 24 h after induction with 10 μM of pravastatin. Results are expressed relatively to untreated samples as mean ± SEM (*n* = 3). Statistical significance was determined by Student’s test, *P < 0.05, ***P < 0.001. **b**, **c** Western blot analyses of the expression of LDLR (**b**) and PCSK9 (**c**) in iHeps treated or not with pravastatin for 24 h or 48 h. The LDLR and PCSK9 bands were quantified with the ImageJ software with normalization by signals of β-actin. Results are expressed relatively to untreated samples as mean ± SEM (*n* = 3). Statistical significance was determined by Student’s test, *P < 0.05, **P < 0.01. **d** Representative confocal images of Dil-LDL internalization in Ctl- and corr-FH-iHeps treated or not with pravastatin for 24 h. Nuclei were counterstained with DAPI. Scale bars: 100 μm. FACS analysis of internalized Dil-LDL levels: results are expressed as mean fluorescence intensity (MFI) relative to untreated samples (*n* = 3). **e** Immunofluorescence staining for SREBP2 in FH-iHeps and in corr-FH-Heps treated or not with pravastatin for 24 h, showing SREBP2 translocation into the nuclei after pravastatin treatment. Nuclei were counterstained with DAPI. Scale bars: 50 μm

Addition of 10 μM of pravastatin to differentiated iHeps for 24 h resulted in a significant induction of HMGCR and PCSK9 mRNA in the 3 cell lines (Fig. 5a)*.* As *SREBP2* regulation is mostly post-transcriptional [1], the level of its transcript was unchanged after pravastatin treatment. At the protein level, LDLR expression was undetectable in FH-iHeps even with pravastatin treatment. By contrast, it was induced 3- and 2.5-fold in control iHeps and in corr-FH-iHeps, respectively (Fig. 5b). This demonstrates that the *APOA2* promoter contained in the therapeutic cassette is functional and can be activated by pravastatin. PCSK9 is synthesized as an inactive zymogen of 72-kDa (proPCSK9) that undergoes intra-molecular autocatalytic cleavage in the endoplasmic reticulum, producing the 60 kDa mature and active form of PCSK9, which is secreted [36]. Both forms were visible and were also induced by pravastatin in both FH-iHeps and corr-FH-iHeps (Fig. 5c). By increasing LDLR expression, pravastatin also increased LDL uptake: 40% in control cells and 20% in corr-FH-iHeps (Fig. 5d)**.** In FH-iHeps and corr-FH-iHeps, SREBP2 was located in extranuclear membranes, whereas in pravastatin-treated cells it was mostly found in the nucleus, consistent with its expected activation and translocation (Fig. 5e). Collectively, our results show that FH-iHeps and corr-FH-iHeps faithfully recapitulate the regulation of genes involved in cholesterol metabolism.

### iHeps support the complete, productive HCV infection cycle, albeit with differences between FH-iHeps and corr-FH-iHeps

**Fig. 6:**
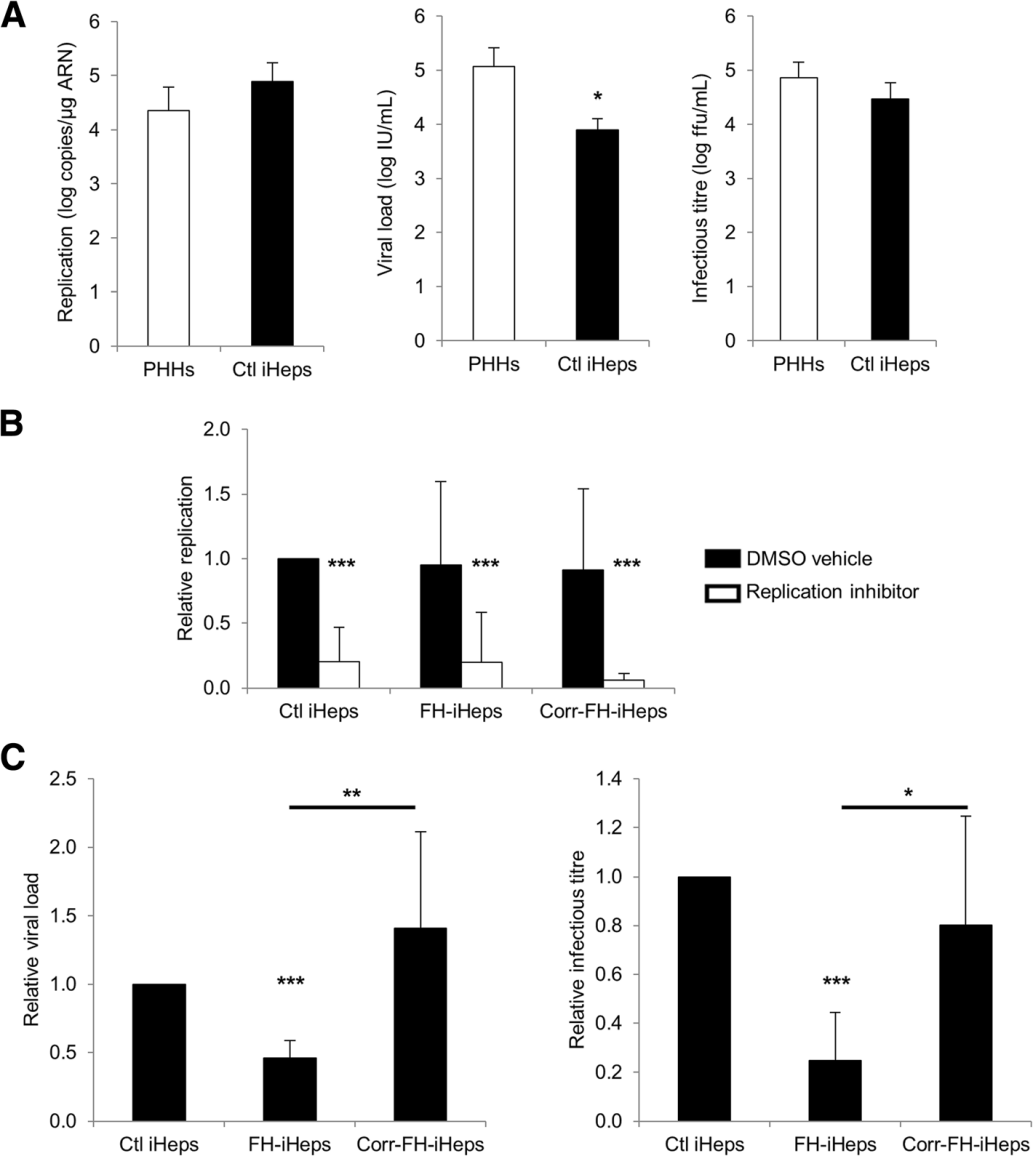
iHeps support the complete HCV infection cycle at levels comparable to PHHs but viral production is decreased in FH-iHeps. **a** Comparison of control (Ctl) iHeps to PHHs for HCV genome replication (intracellular levels of negative-strand HCV RNA) and for production of total (viral load) and infectious (infectious titer) virus. **b** Comparison of Ctl iHeps, FH-iHeps and corr-FH-iHeps for HCV genome replication. Cells were cultured in the presence of 500 nM of the HCV polymerase inhibitor sofosbuvir (replication inhibitor) or dimethylsulfoxide as carrier control (DMSO vehicle). **c** Comparison of Ctl iHeps, FH-iHeps and corr-FH-iHeps for production of total and infectious virus. Results are expressed as mean ± SEM (*n* = 4 to 7 experiments). Statistical significance was determined by Student’s test, *P < 0.05, **P < 0.01, ***P < 0.001

To investigate whether iHeps generated with our differentiation protocol were permissive to HCV infection, we first inoculated control iHeps with JFH1-HCV [18, 24]. We quantified the intracellular levels of negative-strand HCV RNA at day 1 post-inoculation, i.e., under conditions of a single infection cycle, which accurately assess HCV genome replication following virus entry [18, 24]. Not only was this marker detected but its levels were comparably high in iHeps and in PHHs inoculated under identical conditions (Fig. 6a—left panel). The specific HCV polymerase inhibitor sofosbuvir significantly reduced the levels of negative-strand HCV RNA, confirming authentic de novo replication (Fig. 6b). We then used a commercial standardized viral load test and an in-house titration assay to assess the production of total and infectious HCV particles, respectively [18, 24]. Although the mean viral load was 1.17 log lower in iHeps compared to PHHs, the infectious titers were not significantly different and consistently reached 10^4^–10^5^ focus-forming units (ffu)/mL (Fig. 6a—middle and right panels). We conclude that iHeps in our system reproduce the complete HCV life cycle including production of high titers of infectious particles.

We next compared control *LDLR*+/+ iHeps, FH-iHeps and corr-FH-iHeps to reassess the role of the LDLR in the HCV infection cycle. We found that negative-strand HCV RNA was present at equivalent levels in control iHeps, FH-iHeps and corr-FH-iHeps (Fig. 6b), suggesting that neither virus entry nor viral genome replication was affected by the absence of LDLR. Interestingly, however, the viral load and the infectious titer were both significantly decreased, albeit not abolished, in FH-iHeps compared to control iHeps, and restored to normal levels in corr-FH-iHeps (Fig. 6c). Collectively, these data reveal a novel role of LDLR in late steps of the HCV infection cycle, i.e., assembly or secretion of progeny virus.

## Discussion

Few FH homozygotes with a total loss of receptor function survive past age 30 [1]. We report here the first FH-iPSC and FH-iHep models of a FH patient with homozygous null *LDLR* mutation leading to a complete absence and functionality of the LDLR protein. As recently reported [29], current protocols to generate hepatocyte-like cells generally use undefined components in the differentiation process. We designed a novel differentiation approach to generate hepatocytes in conditions based on the use of factors and small molecules close to good manufacturing practices mainly to improve the differentiation of hepatoblasts. Our process resulted in metabolically functional hepatocytes with enhanced hepatocyte polarization and canalicular excretion, suggesting a mature feature of stem cell-derived hepatocytes in vitro.

Recently, CRISPR/Cas9 technology was used to genetically correct iPSCs with a homozygous 3 bp deletion in the exon 4 of the LDLR sequence leading to a weak expression of the protein [37]. We demonstrate here that the strategy based on the targeted insertion of a correcting cassette into the *AAVS1* safe harbor locus via CRISPR/Cas9 can also be used to efficiently restore LDLR phenotype and function. Targeting such a locus rather than the *LDLR* one will allow correcting FH patients’ specific cell lines with distinct mutations using the same therapeutic cassette, as there are more than 1400 mutations known on the *LDLR* gene. It could also be adapted to other hepatic diseases as Hemophilia B or Wilson disease just by swapping the cDNA in the therapeutic cassette.

We also chose the *AAVS1* locus because gene targeting at this site, using different nucleases, showed robust transgene expression in pluripotent stem cells and differentiated progeny [38]. However, hepatic transgene expression in the *AAVS1* context had not yet been established. Indeed, integration into the *AAVS1* locus by means of an FLPe recombinase-mediated cassette exchange of various transgenes under the control of *AFP* enhancer-promoter and *A1AT* promoter resulted in inhibition of transgene expression in transgenic cell lines. This inhibition exerted by the *AAVS1* locus was found both in undifferentiated hESCs and in differentiating hepatocytes [39]. In our construct, 900 bp of specific *APOA2* regulatory sequences drive the expression of LDLR, of which upstream regulatory sequences are not yet fully characterized [40]. They were also chosen because they are specifically expressed in hepatocytes in vitro and in vivo after transplantation of autologous hepatocytes transduced with *APOA2*-GFP lentivectors into cynomolgus macaca livers [41]. Moreover, *APOA2* promoter, like *LDLR* promoter, is regulated by different drugs such as fibrates and statins [35]. We show here that these *APOA2* sequences driving expression of the LDLR can be upregulated by pravastatin, statins being the most used drugs to lower cholesterol. Thus, in a clinical context, our correction strategy makes statin treatment accessible to null-LDLR patients to allow greater cholesterol lowering. The *AAVS1* locus can also be considered for robust and regulated transgene expression in liver, provided that the promoter driving transgene expression contains adequate regulatory sequences. Our study appears to be the first to report the use of this system for genetic correction of a metabolic liver disease with a regulated expression of correcting sequences, both of which being critical for translating the iPSC technology into novel therapies for curing many monogenic disorders. Concerning FH treatment, it has been estimated that as low as 2–5% of hepatic LDLR activity should be sufficient to render homozygous patients accessible to drug treatment [41]. In this clinical context, quality controls by genome sequencing analyses must be done as CRISPR/Cas9 systems could lead to genomic rearrangements [42].

Dyslipidemia is a central causative factor of atherosclerosis. Therefore, the development of appropriate cell models to study cholesterol homeostasis, as well as the action of statins and other pharmaceuticals, is important for cardiovascular health/disease prevention [43]. To address the pathophysiology of FH, we studied the regulation by pravastatin of three genes encoding proteins that regulate cholesterol level and LDLR expression in hepatocytes. Statins are lipid-lowering drugs decreasing intracellular cholesterol levels by direct inhibition of HMGCR, the rate-limiting enzyme in cholesterol biosynthesis. This decrease in intracellular cholesterol level triggers SREBP2 activation that enhances *LDLR* expression increasing LDL uptake by the hepatocytes [1]. SREBP2 also increased *HMGCR* expression which is counteracted by the direct inhibition of HMGCR by statins at the protein level [44]. In our three cell models, as expected, *PCSK9* and *HMGCR* but not *SREBP2* were upregulated at the transcriptional level by pravastatin, which also enhanced expression of PCSK9 and of LDLR—except in FH-iHeps—upon activation of SREBP2. This overall resulted in an increase in Dil-LDL internalization.

FH-patient-specific iPSCs will also be important tools to create autologous controls to elucidate the functional contribution of LDLR mutations to variations that affect cholesterol metabolism and to assess drugs for hypolipidemic functions, which block enzymes at other stages of the cholesterol biosynthetic pathway than statins [44]. Indeed, most drugs that lower serum LDL-c act on the liver through upregulation of *LDLR* transcription (statins) or stability of the LDLR protein by means of PCSK9 inhibitors, such drugs being ineffective in homozygous patients. The need to identify new drugs for the treatment of homozygous FH has recently been elegantly highlighted in vitro and in vivo [45]. We report here a complete LDLR-deficient iHep in vitro model and its autologous genetically corrected counterpart able to recapitulate cholesterol regulation. Both models, in addition to the control *LDLR*+/+, will now be useful to screen drugs in order to develop new therapeutics for homozygous FH.

The use of pluripotent stem cell-derived hepatocytes as an HCV culture model has so far been limited by the low titers of infectious virus produced, most likely attributable to their immature phenotype compared with PHHs [20].

Production of infectious virus necessitates high levels of HCV genome replication but involves additional requirements such as components of the VLDL biogenesis pathway thought to be hijacked by HCV for the formation of lipo-viro-particles [17]. Carpentier et al. noted that their iPSC-derived hepatocyte-like cells secreted APOB-100 (one molecule of which is present in each VLDL) at lower levels than PHHs and also produced low titers of HCV particles (10^2^ ffu/mL) despite significant levels of HCV replication [20]. By contrast, our iHeps secreted APOB-100-containing lipoproteins (LDLR+/+ iHeps: 2.44+/− 0.26 log μg/dL; PHHs: 2.51+/− 0.14 log μg/dL; *P* = 0.30) and produced infectious HCV particles at levels that were both similar to those found in our robust HCV culture system in PHHs [19]. The degree of polarization reached by our iHeps might also contribute to infectious virus production, as recently reported [46]. Thus, we have developed a robust iPSC-based HCV culture system. FH-iHeps and corr-FH-iHeps further provide a proof of concept that these models can be used to study the influence of host genetics on HCV infection and to produce genetically modified hepatocytes that could, for instance, be rendered resistant to viral infection in the context of regenerative medicine. The LDLR has been reported to play a role in the HCV infection cycle, although it may be dispensable and redundant with that of SRBI and of the VLDL receptor [15, 16, 47, 48]. Although this role is generally attributed to the hepatocellular attachment of the lipo-viro-particle via its lipoprotein moiety leading to virus entry [15, 47, 48], Albecka et al. demonstrated that the LDLR-mediated internalization of HCV does not necessarily lead to a productive entry and further revealed an indirect role of the LDLR in HCV genome replication per se via its physiological function in the hepatocellular lipid metabolism [16]. However, these conflicting data were obtained using Huh-7 sublines [16, 47, 48]. Our finding that FH-iHeps supported the complete HCV life cycle despite a complete absence of *LDLR* gene expression confirms that the LDLR is not necessarily required for HCV infection. Nevertheless, the significantly decreased viral load and infectious titer compared to those of both their control and corrected counterparts, despite similar intracellular levels of negative-strand HCV RNA, revealed a novel role of LDLR in late steps of the HCV infection cycle, i.e., assembly or secretion of progeny virus. As the morphogenesis of HCV particles is tightly linked to the lipid metabolism of the producing cell [17, 19], the mechanism underlying the role of LDLR in this process is most likely indirect through the repercussion of lipoprotein uptake on the intracellular lipid content. The availability of FH-iHeps and corr-FH-iHeps provides a valuable model to gain deeper insight into the hepatocellular lipid metabolism and the host requirements for HCV morphogenesis, still the least understood step of the HCV infection cycle.

## Conclusion

In summary, we provide an FH-modeling/therapeutic platform for other FH patients, which can be extrapolated to other inherited liver diseases in order to investigate and rescue defective functions. It also lays the foundation for the development and preclinical assessment of cholesterol-lowering drugs and antiviral therapeutics against human hepatotropic pathogens.

## Additional files

Additional file 1: **Figure S1.** Characterization of FH-iPSCs. (A) FH-iPSCs display the typical hESC-like morphology cultured on irradiated MEFs. (B) Representative RT-PCR for indicated stem cell markers in FH-iPSCs. (C) Representative FACS analyses for indicated stem cell markers in FH-iPSCs. Black lines indicate isotype control antibody and red lines, positive cell immunostaining. (D) Representative pictures of immunostainings for indicated stem cell markers in FH-iPSCs. Scale bars: 50 μm. (E) Karyotype analysis showed no genetic abnormalities induced by the correction process. (F) FH-iPSCs generated embryoid bodies expressing specific proteins of derivatives from the 3 embryonic germ layers: PAX6 (ectoderm), αSMA (mesoderm) and AFP (endoderm). (TIF 2224 kb)

Additional file 2: **Figure S2.** Confirmation of the correct homologous recombination during the insertion of the therapeutic cassette. (A-B) Forward (A) and reverse (B) sequence alignments for the 5′ homology arm between corr-FH-iPSC (lower lane) and the plasmid used for the homologous recombination (upper lane). (C-D) Forward (C) and reverse (D) sequence alignments for the 3′ homology arm between corr-FH-iPSC (lower lane) and the plasmid used for the homologous recombination (upper lane) . (TIF 3705 kb)

Additional file 3: **Figure S3.** Expression of specific stage-markers during the differentiation process. (A) Schematic outline of the developed protocol. (B) Representative RT-PCR analyses at day 0 (hiPSCs), 5 (DE), 10 (hepatoblasts), 18 (pre-hepatocytes) and 25 (iHeps) for expression of stem cells and hepatocytic markers in FH-, corr-FH- and control (Ctl) cells. hESCs were used as positive control for OCT4 and NANOG, and human liver for all others. (TIF 3833 kb)

Additional file 4: **Figure S4.** Differentiation of control, FH- and corr-FH-iPSCs towards definitive endoderm and hepatoblasts. (A) Representative pictures of cell morphology, immunostainings and FACS analyses of the definitive endoderm (DE) markers GATA4, FOXA2 and CXCR4 in cells derived from FH-, corr-FH- and control (Ctl) iPSCs at day 5 of differentiation. Scale bars: 50 μm. (B) Representative pictures of cell morphology, immunostainings and FACS analyses of hepatoblast markers HNF4α, CK19 and EPCAM in cells derived from FH-, corr-FH- and control (Ctl) iPSCs at day 10 of differentiation. Scale bars: 50 μm. (TIF 727 kb)

Additional file 5: **Figure S5.** Differentiation of control (Ctl) iPSCs into hepatocytes. Representative pictures of cell morphology and immunostainings of the indicated markers at day 25 of differentiation. Scale bars: 50 μm. (TIF 4482 kb)

Additional file 6: **Figure S6.** Functionality of differentiated iHeps. (A) Quantitative RT-PCR analyses of APOA1, APOB, APOC3 and APOE at day 25 of differentiation in control (Ctl) iHeps, corr-FH-iHeps and FH-iHeps. Data are represented as the percentage of expression in HepG2 hepatocytic cell line. (B) Quantitative RT-PCR analyses of 3 major CYPs, CYP2D6, CYP3A4, and CYP2A6 at day 25 of differentiation in Ctl iHeps, corr-FH-iHeps and FH-iHeps. Data are represented as the percentage of expression in HepG2 hepatocytic cell line. (C) Albumin secretion by Ctl, FH- and corr-FH-iHeps was determined by ELISA test. (D) Oil red staining shows the ability of cells to store lipids. Scale bars: 200 μm. (E) Periodic Acid Schiff staining shows the ability of cells to store glycogen. Scale bars: 200 μm (F) Uptake and excretion of Indocyanin Green (ICG). After 1 h of incubation, the uptake of ICG was visible in a high percentage of FH- and corr-FH-iHeps. Most of these cells had excreted ICG as early as 4 h after its withdrawal from the medium. Scale bars: 200 μm. (TIF 2044 kb)

## Data Availability

Data sharing is not applicable to this article as no datasets were generated or analyzed during the current study.

## Abbreviations

AAVS1: Adeno-associated virus integration site 1
APOA2: Apolipoprotein A2
APOB-100: Apolipoprotein B100
Cas: CRISPR-associated system
CRISPR: Clustered regularly interspaced short palindromic repeats
Dil-LDL: 1,1′-Dioctadecyl-3,3,3,3′-tetramethylindocarbocyanineperchlorate
FH: Familial hypercholesterolemia
HCV: Hepatitis C virus
HMGCR: 3-Hydroxy-3-methyl-glutaryl-coenzyme A reductase
iHeps: iPSC-derived hepatocytes
iPSCs: Induced pluripotent stem cells
LDL-c: Low-density lipoprotein cholesterol
LDLR: Low-density lipoprotein receptor
PCSK9: Proprotein convertase subtilisin kexin/type 9
PHH: Primary human hepatocytes
SREBP: Sterol regulatory element-binding protein
VLDL: Very-low-density lipoprotein
