# Supplementary figures and images for: Low-density lipoprotein receptor-deficient hepatocytes differentiated from induced pluripotent stem cells allow familial hypercholesterolemia modeling, CRISPR/Cas-mediated genetic correction, and productive hepatitis C virus infection
Source: Stem Cell Res Ther. 2019 Jul 29;10:221. doi: 10.1186/s13287-019-1342-6 (PMC6664765; doi:10.1186/s13287-019-1342-6)

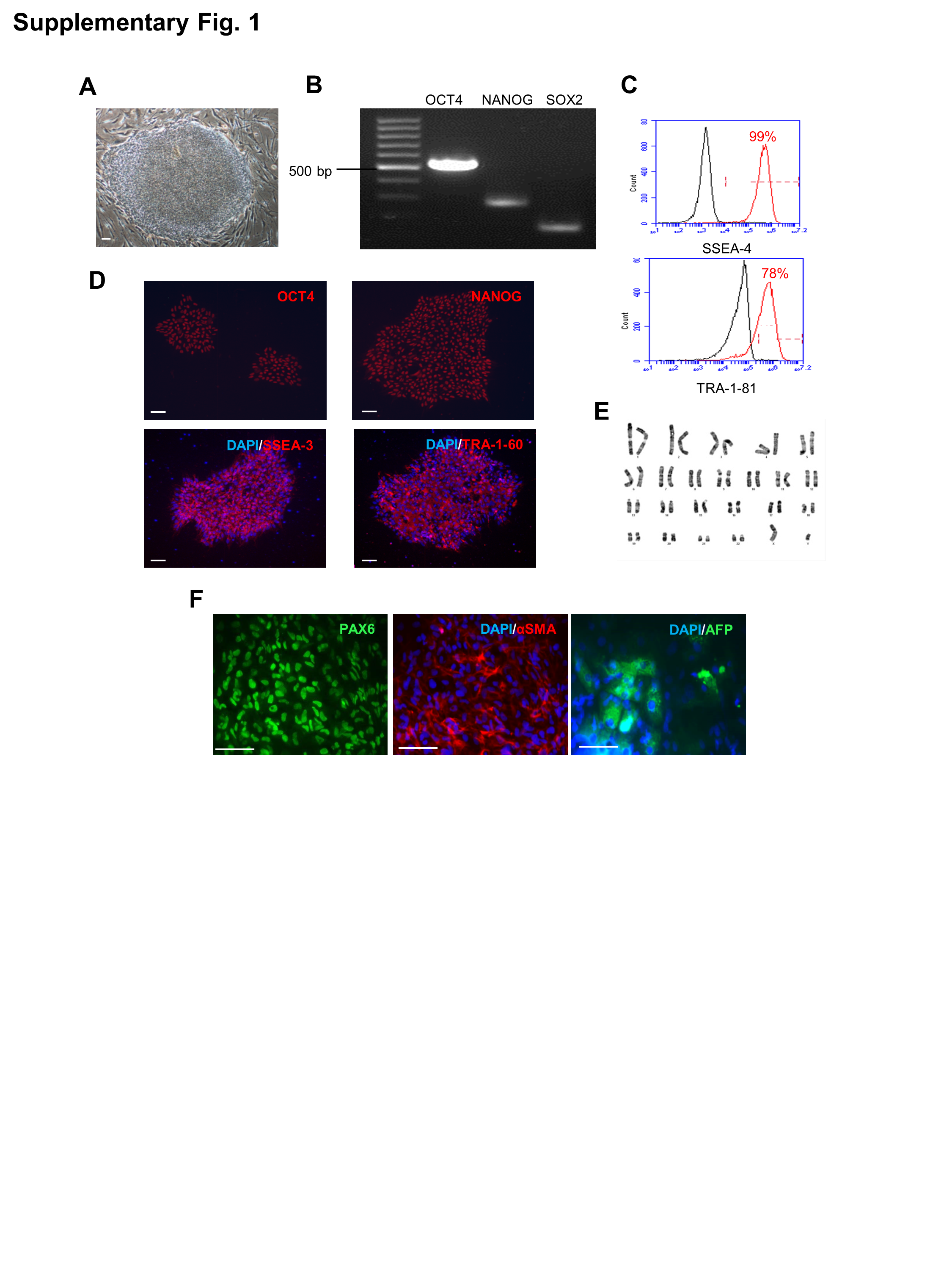

Supplement: Supplementary file 1 — Figure S1. Characterization of FH-iPSCs. (A) FH-iPSCs display the typical hESC-like morphology cultured on irradiated MEFs. (B) Representative RT-PCR for indicated stem cell markers in FH-iPSCs. (C) Representative FACS analyses for indicated stem cell markers in FH-iPSCs. Black lines indicate isotype control antibody and red lines, positive cell immunostaining. (D) Representative pictures of immunostainings for indicated stem cell markers in FH-iPSCs. Scale bars: 50 μm. (E) Karyotype analysis showed no genetic abnormalities induced by the correction process. (F) FH-iPSCs generated embryoid bodies expressing specific proteins of derivatives from the 3 embryonic germ layers: PAX6 (ectoderm), αSMA (mesoderm) and AFP (endoderm). (TIF 2224 kb) [file 13287_2019_1342_MOESM1_ESM.tif]

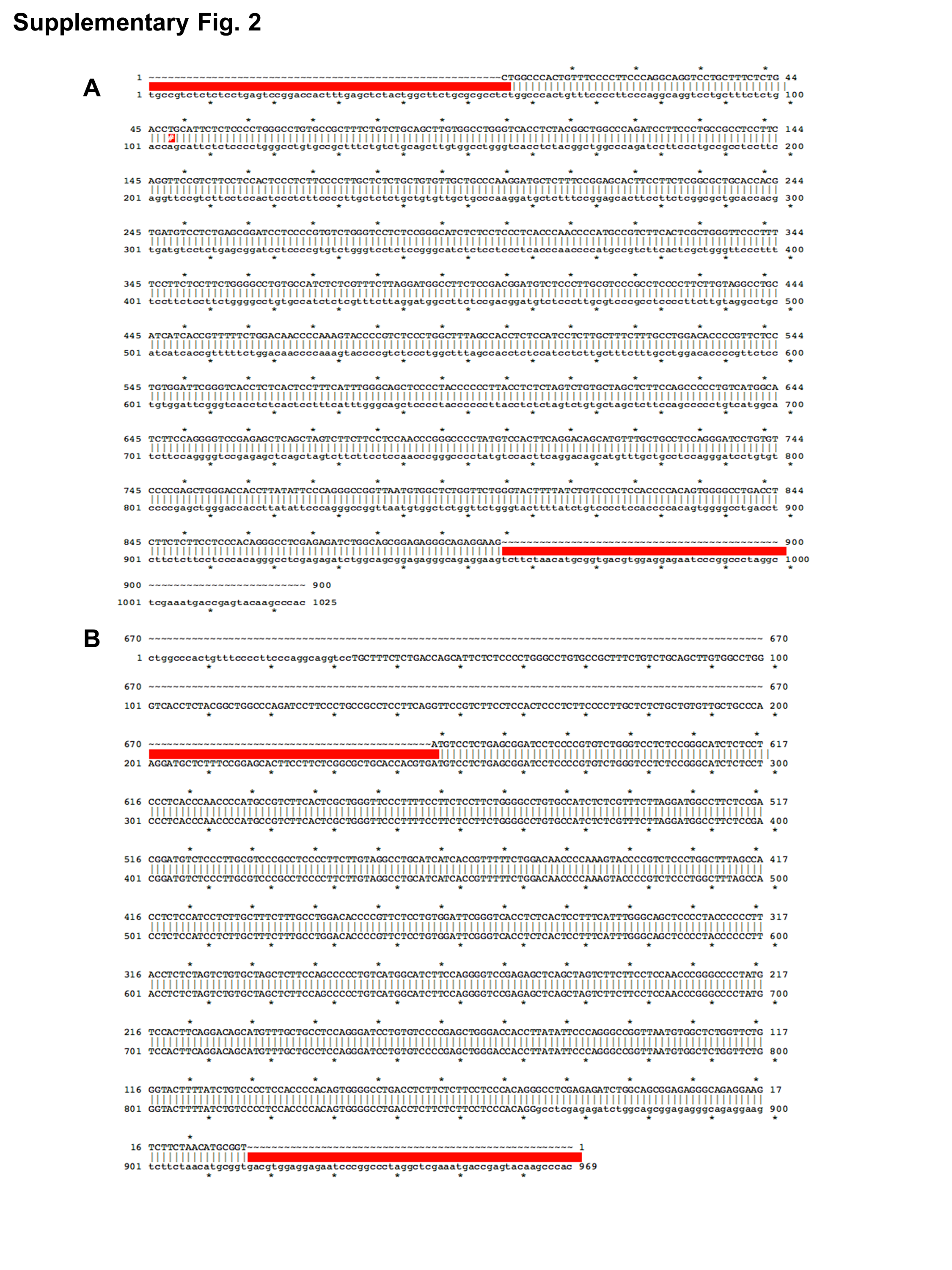

Supplement: Supplementary file 2 — Figure S2. Confirmation of the correct homologous recombination during the insertion of the therapeutic cassette. (A-B) Forward (A) and reverse (B) sequence alignments for the 5′ homology arm between corr-FH-iPSC (lower lane) and the plasmid used for the homologous recombination (upper lane). (C-D) Forward (C) and reverse (D) sequence alignments for the 3′ homology arm between corr-FH-iPSC (lower lane) and the plasmid used for the homologous recombination (upper lane) . (TIF 3705 kb) [file 13287_2019_1342_MOESM2_ESM.tif]

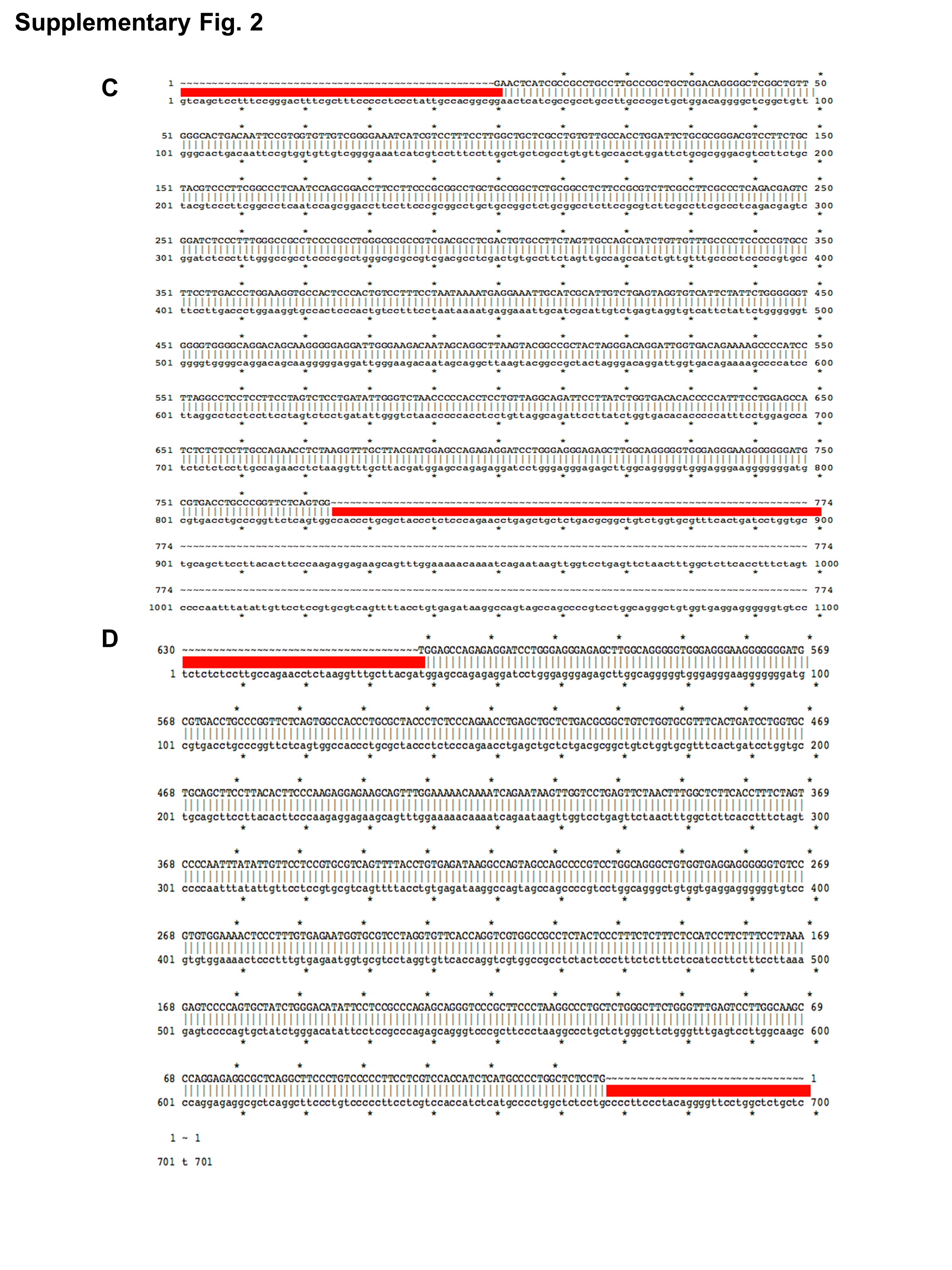

Supplement: Supplementary file 3 — Figure S3. Expression of specific stage-markers during the differentiation process. (A) Schematic outline of the developed protocol. (B) Representative RT-PCR analyses at day 0 (hiPSCs), 5 (DE), 10 (hepatoblasts), 18 (pre-hepatocytes) and 25 (iHeps) for expression of stem cells and hepatocytic markers in FH-, corr-FH- and control (Ctl) cells. hESCs were used as positive control for OCT4 and NANOG, and human liver for all others. (TIF 3833 kb) [file 13287_2019_1342_MOESM3_ESM.tif]

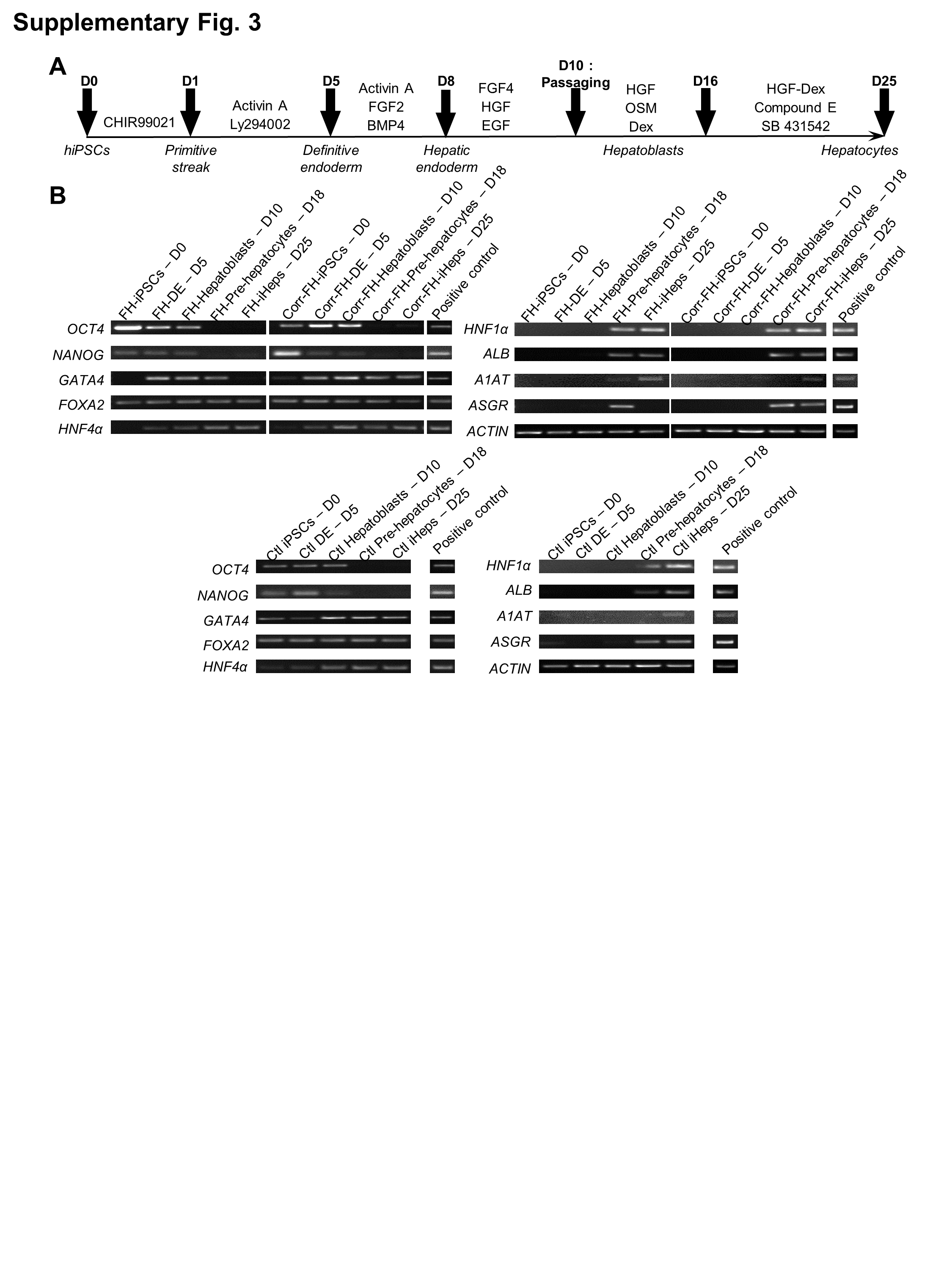

Supplement: Supplementary file 4 — Figure S4. Differentiation of control, FH- and corr-FH-iPSCs towards definitive endoderm and hepatoblasts. (A) Representative pictures of cell morphology, immunostainings and FACS analyses of the definitive endoderm (DE) markers GATA4, FOXA2 and CXCR4 in cells derived from FH-, corr-FH- and control (Ctl) iPSCs at day 5 of differentiation. Scale bars: 50 μm. (B) Representative pictures of cell morphology, immunostainings and FACS analyses of hepatoblast markers HNF4α, CK19 and EPCAM in cells derived from FH-, corr-FH- and control (Ctl) iPSCs at day 10 of differentiation. Scale bars: 50 μm. (TIF 727 kb) [file 13287_2019_1342_MOESM4_ESM.tif]

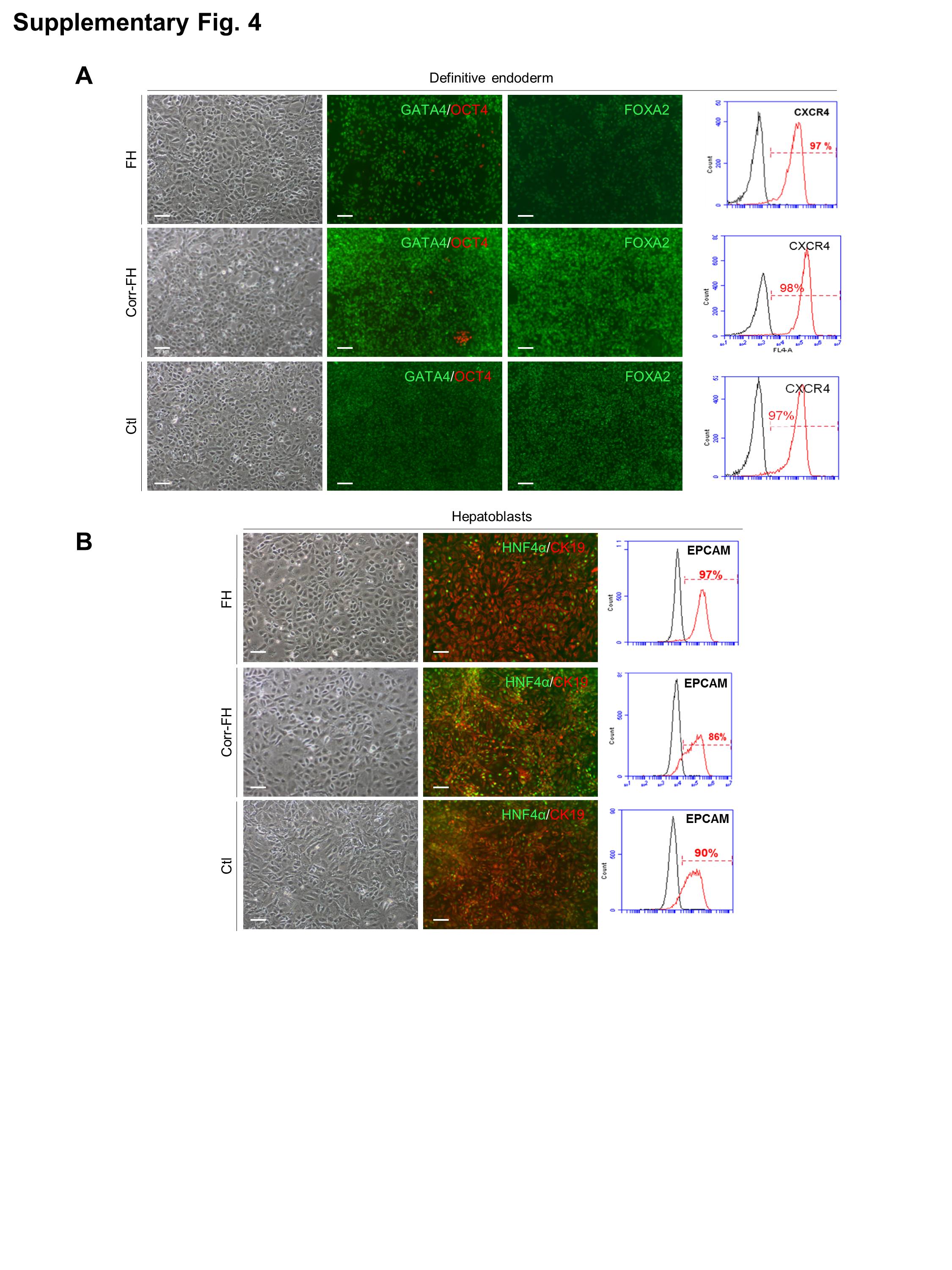

Supplement: Supplementary file 5 — Figure S5. Differentiation of control (Ctl) iPSCs into hepatocytes. Representative pictures of cell morphology and immunostainings of the indicated markers at day 25 of differentiation. Scale bars: 50 μm. (TIF 4482 kb) [file 13287_2019_1342_MOESM5_ESM.tif]

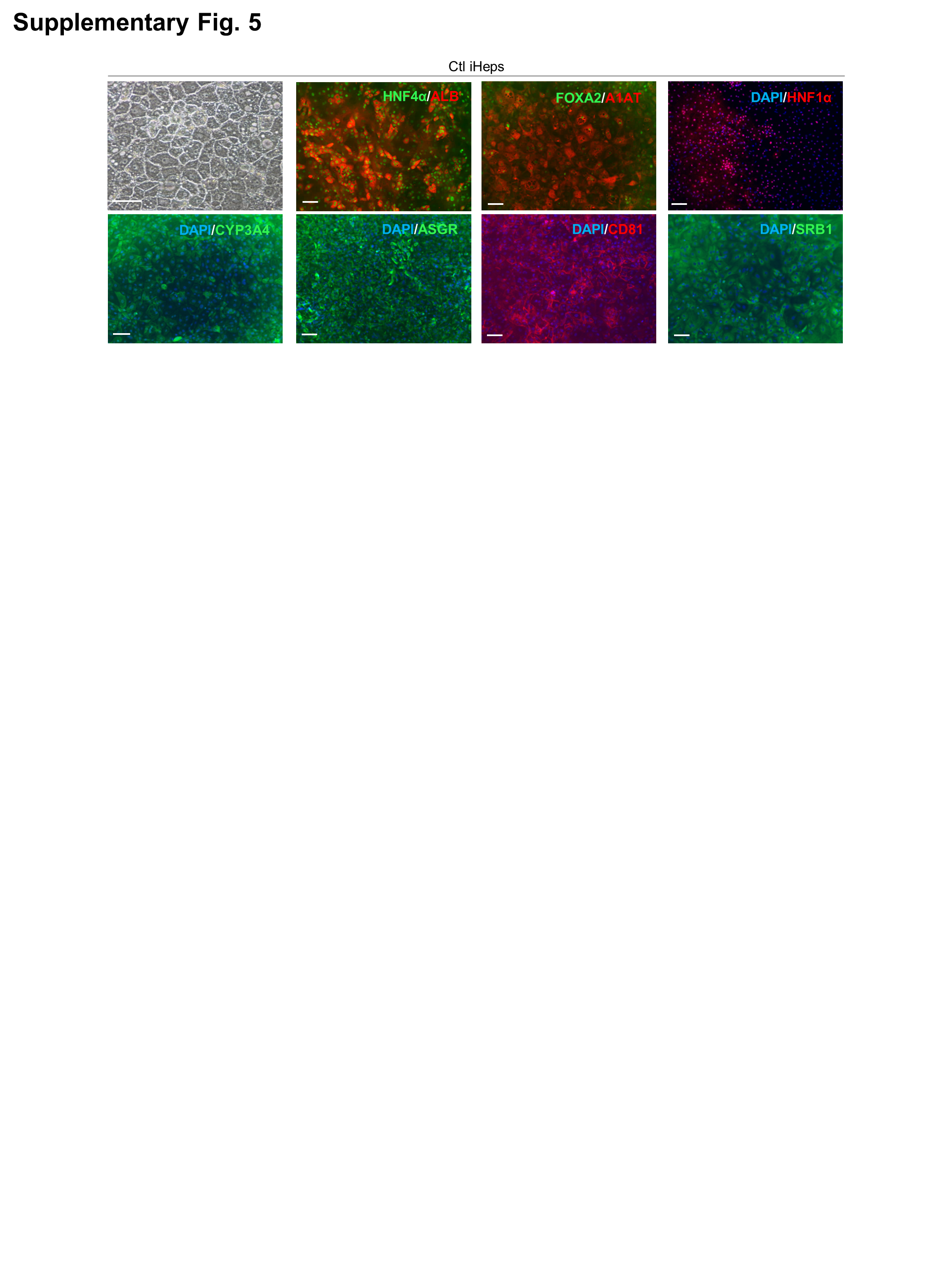

Supplement: Supplementary file 6 — Figure S6. Functionality of differentiated iHeps. (A) Quantitative RT-PCR analyses of APOA1, APOB, APOC3 and APOE at day 25 of differentiation in control (Ctl) iHeps, corr-FH-iHeps and FH-iHeps. Data are represented as the percentage of expression in HepG2 hepatocytic cell line. (B) Quantitative RT-PCR analyses of 3 major CYPs, CYP2D6, CYP3A4, and CYP2A6 at day 25 of differentiation in Ctl iHeps, corr-FH-iHeps and FH-iHeps. Data are represented as the percentage of expression in HepG2 hepatocytic cell line. (C) Albumin secretion by Ctl, FH- and corr-FH-iHeps was determined by ELISA test. (D) Oil red staining shows the ability of cells to store lipids. Scale bars: 200 μm. (E) Periodic Acid Schiff staining shows the ability of cells to store glycogen. Scale bars: 200 μm (F) Uptake and excretion of Indocyanin Green (ICG). After 1 h of incubation, the uptake of ICG was visible in a high percentage of FH- and corr-FH-iHeps. Most of these cells had excreted ICG as early as 4 h after its withdrawal from the medium. Scale bars: 200 μm. (TIF 2044 kb) [file 13287_2019_1342_MOESM6_ESM.tif]
